# Cartilage-specific *Sirt6* deficiency represses IGF-1 and enhances osteoarthritis severity in mice

**DOI:** 10.1136/ard-2023-224385

**Published:** 2023-08-07

**Authors:** John A Collins, C James Kim, Ashley Coleman, Abreah Little, Matheus M Perez, Emily J Clarke, Brian Diekman, Mandy J Peffers, Susanna Chubinskaya, Ryan E Tomlinson, Theresa A Freeman, Richard F Loeser

**Affiliations:** 1 Department of Orthopaedic Surgery, Thomas Jefferson University, Philadelphia, Pennsylvania, USA; 2 Department of Medicine, Division of Rheumatology, Allergy and Immunology and the Thurston Arthritis Research Center, The University of North Carolina at Chapel Hill, Chapel Hill, North Carolina, USA; 3 Institute of Life Course and Medical Sciences, University of Liverpool, Liverpool, UK; 4 Department of Pediatrics, Rush University Medical Center, Chicago, Illinois, USA

**Keywords:** osteoarthritis, chondrocytes, arthritis, experimental, osteoarthritis, knee, biological therapy

## Abstract

**Objectives:**

Prior studies noted that chondrocyte SIRT6 activity is repressed in older chondrocytes rendering cells susceptible to catabolic signalling events implicated in osteoarthritis (OA). This study aimed to define the effect of *Sirt6* deficiency on the development of post-traumatic and age-associated OA in mice.

**Methods:**

Male cartilage-specific *Sirt6*-deficient mice and *Sirt6* intact controls underwent destabilisation of the medial meniscus (DMM) or sham surgery at 16 weeks of age and OA severity was analysed at 6 and 10 weeks postsurgery. Age-associated OA was assessed in mice aged 12 and 18 months of age. OA severity was analysed by micro-CT, histomorphometry and scoring of articular cartilage structure, toluidine blue staining and osteophyte formation. SIRT6-regulated pathways were analysed in human chondrocytes by RNA-sequencing, qRT-PCR and immunoblotting.

**Results:**

*Sirt6-*deficient mice displayed enhanced DMM-induced OA severity and accelerated age-associated OA when compared with controls, characterised by increased cartilage damage, osteophyte formation and subchondral bone sclerosis. In chondrocytes, RNA-sequencing revealed that *SIRT6* depletion significantly repressed cartilage extracellular matrix (eg, *COL2A1*) and anabolic growth factor (eg, insulin-like growth factor-1 (*IGF-1*)) gene expression. Gain-of-function and loss-of-function studies in chondrocytes demonstrated that SIRT6 depletion attenuated, whereas adenoviral overexpression or MDL-800-induced *SIRT6* activation promoted IGF-1 signalling by increasing Akt^ser473^ phosphorylation.

**Conclusions:**

SIRT6 deficiency increases post-traumatic and age-associated OA severity in vivo. SIRT6 profoundly regulated the pro-anabolic and pro-survival IGF-1/Akt signalling pathway and suggests that preserving the SIRT6/IGF-1/Akt axis may be necessary to protect cartilage from injury-associated or age-associated OA. Targeted therapies aimed at increasing SIRT6 function could represent a novel strategy to slow or stop OA.

**WHAT IS ALREADY KNOWN ON THIS TOPIC**

- Sirt6 activity significantly declines in ageing chondrocytes, which promotes catabolic signalling events implicated in osteoarthritis (OA) development and progression.
- Sirt6 regulates multiple pathways necessary for chondrocyte homeostasis but the effect of Sirt6 deficiency on OA development in vivo and the specific Sirt6-associated mechanisms responsible remain largely unexplored.

**WHAT THIS STUDY ADDS**

- Cartilage-specific Sirt6 deficiency enhances post-traumatic OA and accelerates age-associated OA in mice.
- Depletion of chondrocyte Sirt6 significantly represses insulin-like growth factor-1 (IGF-1) signalling and downregulates multiple cartilage extracellular matrix components including COL2A1.
- Genetic and pharmacological activation of Sirt6 promotes pro-survival and pro-anabolic IGF-1/Akt activation in human chondrocytes.

**HOW THIS STUDY MIGHT AFFECT RESEARCH, PRACTICE OR POLICY**

- The SIRT6/IGF-1 signalling axis is an important mediator of cartilage integrity and the chondrocyte phenotype.
- Targeted therapies that promote chondrocyte SIRT6 activity during ageing and in response to injury represents a novel strategy to reduce OA severity.

## Introduction

The highly conserved NAD^+^-dependent family of sirtuin deacetylases and mono-ADP ribosyltransferases (sirtuins 1–7) are key epigenetic regulators that control age-associated cell signalling pathways and promote longevity in various model organisms.^1 2^ Efforts to elucidate the precise roles of the nuclear localised sirtuin 6 (SIRT6) in ageing and disease have come to the fore since the finding that global loss of *Sirt6* in mice leads to a progeroid phenotype, metabolic dysfunction and death within 4 weeks of birth.^3^ Conversely, transgenic overexpression of *Sirt6* governs metabolic signalling events during ageing to extend lifespan in both male and female mice.^4–6^ Several lines of evidence demonstrate that SIRT6 regulates an array of age-associated biological processes including DNA repair, cellular metabolism, oxidative stress, inflammation, autophagy and senescence.^1 2^ As such, maintenance of SIRT6 activity during ageing, or in response to stress, is considered important for the prevention of ageing diseases such as cardiovascular disease, various metabolic and neurodegenerative disorders including diabetes and Alzheimer’s, certain cancers and arthritis.^1 2 7 8^

Age and joint injury are key risk factors for osteoarthritis (OA), which is the most common form of joint disease and a major cause of disability in the elderly.^9 10^ Age-associated or injury-associated alterations that favour catabolic over anabolic signalling events in chondrocytes promote loss of extracellular matrix (ECM) components and are postulated to drive cartilage degradation in OA development and progression.^9 10^ Recent evidence suggests that SIRT6 may be a critical regulator of these processes.^7 8 11 12^ For example, in vitro cell culture studies have shown that SIRT6 overexpression decreases replicative senescence, matrix metalloproteinase-13 (MMP-13) levels and nuclear factor kappa B (NF-ĸB)-regulated gene expression in human chondrocytes,^11^ whereas SIRT6 depletion increases markers of DNA damage and telomere dysfunction-induced foci in chondrocytes^12^ and significantly represses *COL2A1* and *ACAN* gene expression in the chondrosarcoma SW1353 cell line.^13^ In vivo data in mice demonstrate that *Sirt6* haploinsufficiency increases cartilage proteoglycan loss and infrapatellar fat pad cytokine levels, resulting in higher Osteoarthritis Research Society International (OARSI) scores in middle-aged mice on a high fat diet.^14^ In addition, myeloid-specific *Sirt6* deficiency in mice has been shown to increase joint inflammation and sensitivity to pro-catabolic FOXO1 signalling events to enhance joint inflammation in collagen-induced and K/BxN serum transfer models of rheumatoid arthritis.^15^ Conversely, intra-articular administration of adeno-associated virus or lentiviral SIRT6, to increase SIRT6 levels within the joint space, provides protection against cartilage damage in young mice receiving destabilisation of the medial meniscus (DMM) surgery.^7 11^

Our previous study in primary human chondrocytes demonstrated that activation of SIRT6 promotes resistance to oxidative stress via increasing antioxidant protein levels, decreasing pro-oxidant TXNIP levels and rapidly detoxifying nuclear generated H_2_O_2_.^8^ In addition, activating SIRT6 significantly reduced oxidative stress-induced catabolic NF-ĸB signalling events that are implicated in chondrocyte cell death and OA.^8^ Importantly, our report also showed that chondrocyte SIRT6 activity significantly declines with age in human articular chondrocytes.^8^ The effect of SIRT6 deficiency within the joint, and how this could contribute to cartilage damage and OA in vivo, however, remain largely unexplored. As prior studies investigating the role of *Sirt6* in vivo have used small numbers of experimental mice (n=4–6),^7 11^ the aim of this study was to comprehensively define the effects of *Sirt6* deficiency on OA. As injury and age represent two major risk factors for OA, we examined the effect of *Sirt6* deficiency on younger mice given destabilisation of the medial meniscus surgery as a model of post-traumatic OA, and also assessed spontaneous, naturally occurring OA severity in middle-aged (12 months of age) and older (18 months of age) mice. The specific mechanisms by which SIRT6 regulates chondrocyte function to protect cartilage from OA was examined in these mice as well as in vitro using primary human chondrocytes.

## Materials and methods

Detailed experimental procedures and analyses are provided in online supplemental file.

10.1136/ard-2023-224385.supp1 Supplementary data

## Results

### Cartilage-specific *Sirt6* deficiency increases DMM-induced OA severity in mice

**Figure 1:**
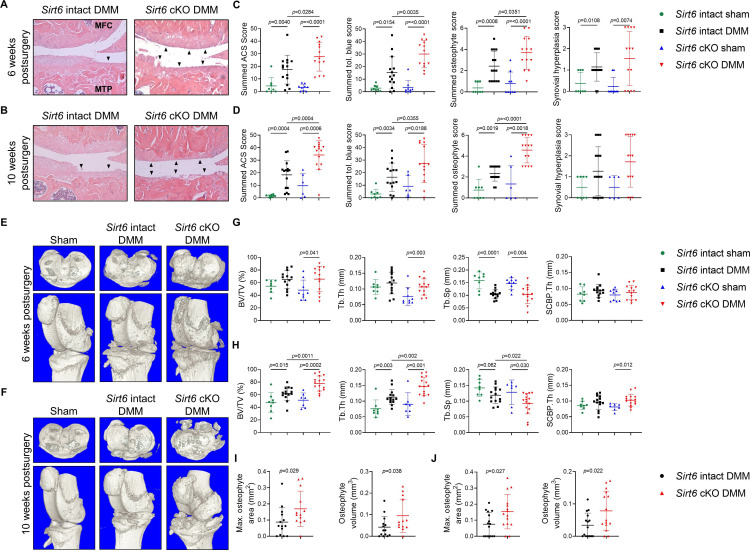
The effect of *Sirt6* deficiency on OA severity after DMM surgery. *Sirt6* intact and *Sirt6* deficient (*Sirt6* cKO) mice received sham or DMM surgery at 16 weeks of age and OA severity was analysed at 6 and 10 weeks post-DMM surgery by histology and micro-CT. (A,B) Representative images of H&E stained midcoronal sections showing the MTP and MFC from *Sirt6* intact and *Sirt6* cKO mice at 6 and 10 weeks post-DMM surgery. (C,D) Summed (MTP, MFC, LTP, LFC) ACS and toluidine blue scores, summed (MTP, LTP) osteophyte scores and synovial hyperplasia scores (medial side) at 6 and 10 weeks post-DMM surgery. Black arrows indicate areas of complete articular cartilage loss. (E–F) Representative three-dimensional micro-CT reconstructions of knee joints from representative sham, *Sirt6* intact and *Sirt6* cKO mice at 6 and 10 weeks post-DMM surgery. Upper panels show transverse images of the tibial plateau and lower panels show images of the whole joint. (G–H) Micro-CT analysis of subchondral bone changes (BV/TV, Tb.Th, Tb.Sp, SCBP.Th) on the medial tibial plateaus of *Sirt6* intact and *Sirt6* cKO mice (DMM and sham groups) at 6 and 10 weeks post-DMM surgery. (I) Micro-CT analysis of max osteophyte area and osteophyte volume on the MTP of *Sirt6* intact and *Sirt6* cKO mice (DMM groups only) at 6 weeks and (J) 10 weeks post-DMM surgery. Individual data points are presented with mean±SD. Significant differences between groups were detected by Mann-Whitney U test comparing sham and DMM groups for each genotype (A–H) or t-test (I,J). Exact p values are presented. ACS, articular cartilage structure; cKO, *Sirt6*-deficient; DMM, destabilisation of the medial meniscus; LFC, lateral femoral condyle; LTP, lateral tibial plateau; MFC, medial femoral condyle; MTP, medial tibial plateau; OA, osteoarthritis; SCBP, subchondral bone plate.

As global loss of *Sirt6* in mice results in death within 4 weeks of age,^3^ we generated inducible cartilage-specific *Sirt6-*deficient mice (*Sirt6^fl/fl^;Aggrecan-Cre^ERT2^*, (*Sirt6* cKO)) and compared them with *Sirt6* intact littermate controls (*Sirt6^fl/fl^*). *Sirt6* intact and *Sirt6*-deficient mice underwent DMM surgery or sham surgery^16^ at 16 weeks of age and OA severity was analysed by histology, detailed histomorphometry and micro-CT at 6 and 10 weeks postsurgery. We have previously demonstrated cartilage SIRT6 deficiency in this model ex vivo,^8^ which has been validated here by immunohistochemistry (IHC) (online supplemental figure 1). Histologically, both *Sirt6* intact and *Sirt6-*deficient mice receiving DMM surgery developed signs of OA, characterised by significant increases in summed articular cartilage structure (ACS), toluidine blue, osteophyte and synovial hyperplasia scores when compared with sham controls at both time points studied (figure 1A–D, online supplemental figures 2 and 3). When comparing DMM groups (*Sirt6* intact vs *Sirt6* cKO), ACS, toluidine blue and osteophyte scores were significantly higher (worse) in *Sirt6-*deficient mice when compared with *Sirt6* intact mice at 6 and 10 weeks postsurgery (figure 1A–D, online supplemental figures 2 and 3).

In alignment with our histological data, detailed histomorphometric analysis of the medial and lateral tibial plateaus showed that mice receiving sham surgery displayed little signs of OA whereas DMM surgery produced an OA phenotype at both time points studied, as evidenced by significant cartilage loss, and in some cases, loss of calcified cartilage and increased subchondral bone plate (SCBP) area and thickness (tables 1 and 2, online supplemental tables 2 and 3). At 6 weeks postsurgery, *Sirt6*-deficient mice receiving DMM surgery displayed significant reductions in articular cartilage area and thickness when compared with *Sirt6* intact mice receiving DMM surgery (table 1, online supplemental table 2). At 10 weeks, this OA phenotype was exacerbated and mice with cartilage-specific *Sirt6* deficiency also displayed significant reductions in calcified cartilage thickness and enhanced SCBP area and thickness when compared with *Sirt6* intact mice undergoing DMM (table 2, online supplemental table 3).

**Table 1:** Histomorphometric analysis of *Sirt6* intact and *Sirt6* deficient (*Sirt6* cKO) mice receiving DMM or sham surgery 6 weeks post-DMM surgery

Parameter	Sirt6 intact sham Mean (SD)	Sirt6 intact DMM Mean (SD)	Sirt6 cKO sham Mean (SD)	Sirt6 cKO DMM Mean (SD)	Sirt6 intact sham versus Sirt6 intact DMM P value	Sirt6 cKO sham versus Sirt6 cKO DMM P value	Sirt6 intact DMM versus Sirt6 cKO DMM P value
Art. Cart. area (µm^2^)	46 566 (12 474)	36 953 (9587)	51 587 (15 812)	24 545 (14 379)	**0.0478**	**0.0005**	**0.0136**
Art. Cart. thickness (µm)	47.15 (10.56)	34.76 (9.88)	48.42 (12.60)	17.60 (11.01)	**0.0121**	**<0.0001**	**0.0003**
Calc. Cart. area (µm^2^)	43 407 (8996)	43 694 (10 658)	37 507 (6625)	53 603 (14 998)	0.9497	**0.0070**	0.0576
Calc. Cart. thickness (µm)	41.95 (7.61)	38.55 (7.08)	42.45 (7.04)	34.60 (7.64)	0.3045	**0.0240**	0.1755
SCBP area (µm^2^)	67 833 (32 720)	83 472 (22 262)	52 673 (17 378)	86 972 (18 713)	0.1963	**0.0003**	0.7723
SCBP thickness (µm)	58.83 (27.6)	59.94 (16.88)	52.96 (18.53)	94.49 (28.81)	0.9077	**0.0011**	**0.0112**

Histomorphometry measurements of Art. Cart. thickness and area, Calc. Cart. thickness and area and SCBP thickness and area were analysed from midcoronal sections of mouse limbs (medial tibial plateau) from *Sirt6* intact and cKO mice receiving DMM or sham surgery 6 weeks post-DMM surgery. *Sirt6* intact sham: n=8; *Sirt6* intact DMM: n=14; *Sirt6* cKO sham: n=9; *Sirt6* cKO DMM: n=13. Results are presented as mean±SD. Significant differences between groups were detected by Mann-Whitney U test comparing sham and DMM groups for each genotype. Exact p values are presented.

Significance values are in bold.

Art. Cart., articular cartilage; Calc. Cart., calcified cartilage; cKO, *Sirt6*-deficient; DMM, destabilisation of the medial meniscus; SCBP, subchondral bone plate.

**Table 2:** Histomorphometric analysis of *Sirt6* intact and *Sirt6* deficient (*Sirt6* cKO) mice receiving DMM or sham surgery 10 weeks post-DMM surgery

Parameter	Sirt6 intact sham Mean (SD)	Sirt6 intact DMM Mean (SD)	Sirt6 cKO sham Mean (SD)	Sirt6 cKO DMM Mean (SD)	Sirt6 intact sham versus Sirt6 intact DMM P value	Sirt6 cKO sham versus Sirt6 cKO DMM P value	Sirt6 intact DMM versus Sirt6 cKO DMM P value
Art. Cart. area (µm^2^)	50 027 (7337)	36 080 (12 399)	43 670 (10 566)	15 334 (12 933)	**0.0078**	**0.0002**	**0.0001**
Art. Cart. thickness (µm)	46.47 (5.41)	30.87 (9.83)	36.43 (6.52)	12.88 (11.27)	**0.0005**	**0.0002**	**<0.0001**
Calc. Cart. area (µm^2^)	57 312 (16 946)	56 891 (7969)	60 243 (6928)	45 417 (22 057)	0.9348	0.1290	0.0686
Calc. Cart. thickness (µm)	44.58 (9.50)	46.66 (8.31)	47.60 (3.87)	35.73 (16.33)	0.5904	0.1000	**0.0296**
SCBP area (µm^2^)	66 156 (22 032)	95 316 (23 622)	79 749 (30 296)	127 842 (36 678)	**0.0089**	**0.0115**	**0.0081**
SCBP thickness (µm)	49.37 (12.37)	76.01 (19.7)	55.14 (9.53)	103.10 (36.47)	**0.0024**	**0.0058**	**0.0185**

Histomorphometry measurements of Art. Cart. thickness and area, Calc. Cart. thickness and area and SCBP thickness and area were analysed from midcoronal sections of mouse limbs (medial tibial plateau) from *Sirt6* intact and cKO mice receiving DMM or sham surgery 10 weeks post-DMM surgery. *Sirt6* intact sham: n=8; *Sirt6* intact DMM: n=15; *Sirt6* cKO sham: n=6; *Sirt6* cKO DMM: n=14. Results are presented as mean±SD. Significant differences between groups were detected by Mann-Whitney U test comparing sham and DMM groups for each genotype. Exact p values are presented.

Significance values are in bold.

Art. Cart., articular cartilage; Calc. Cart., calcified cartilage; cKO, *Sirt6*-deficient; DMM, destabilisation of the medial meniscus; SCBP, subchondral bone plate.

Micro-CT analysis of tibial subchondral bone volume fraction (BV/TV), Trabecular Thickness (Tb.Th), Trabecular Separation (Tb.Sp) and Subchondral Bone Plate thickness (SCBP).Th was conducted on the medial tibial plateaus of all mice. At 6 weeks postsurgery, *Sirt6*-deficient mice receiving DMM surgery exhibited significant increases in BV/TV and Tb.Th and significantly lower Tb.Sp values, indicating enhanced bone sclerosis in this group when compared with *Sirt6*-deficient mice receiving sham surgery (figure 1E and G). No changes were observed between *Sirt6* intact mice from either surgery group at this time point. At 10 weeks postsurgery, both DMM groups displayed increases in BV/TV and Tb.Th and significantly lower Tb.Sp when compared with sham mice, indicating DMM-induced bone sclerosis at this time point (figure 1F and H). When analysing differences between DMM groups, *Sirt6*-deficient mice displayed significant increases in BV/TV and Tb.Th, and reductions in Tb.Sp when compared with *Sirt6* intact mice, suggesting enhanced bone sclerosis in the absence of *Sirt6* (figure 1F and H). Osteophyte area and osteophyte volume were also significantly greater in *Sirt6*-deficient mice receiving DMM surgery when compared with *Sirt6* intact mice (figure 1I,J), which aligns with our histological osteophyte scoring. Collectively, these data demonstrate that *Sirt6* deficiency in mouse cartilage increases the severity of DMM-induced OA.

### Cartilage-specific *Sirt6* deficiency accelerates spontaneous age-associated OA severity in mice

**Figure 2:**
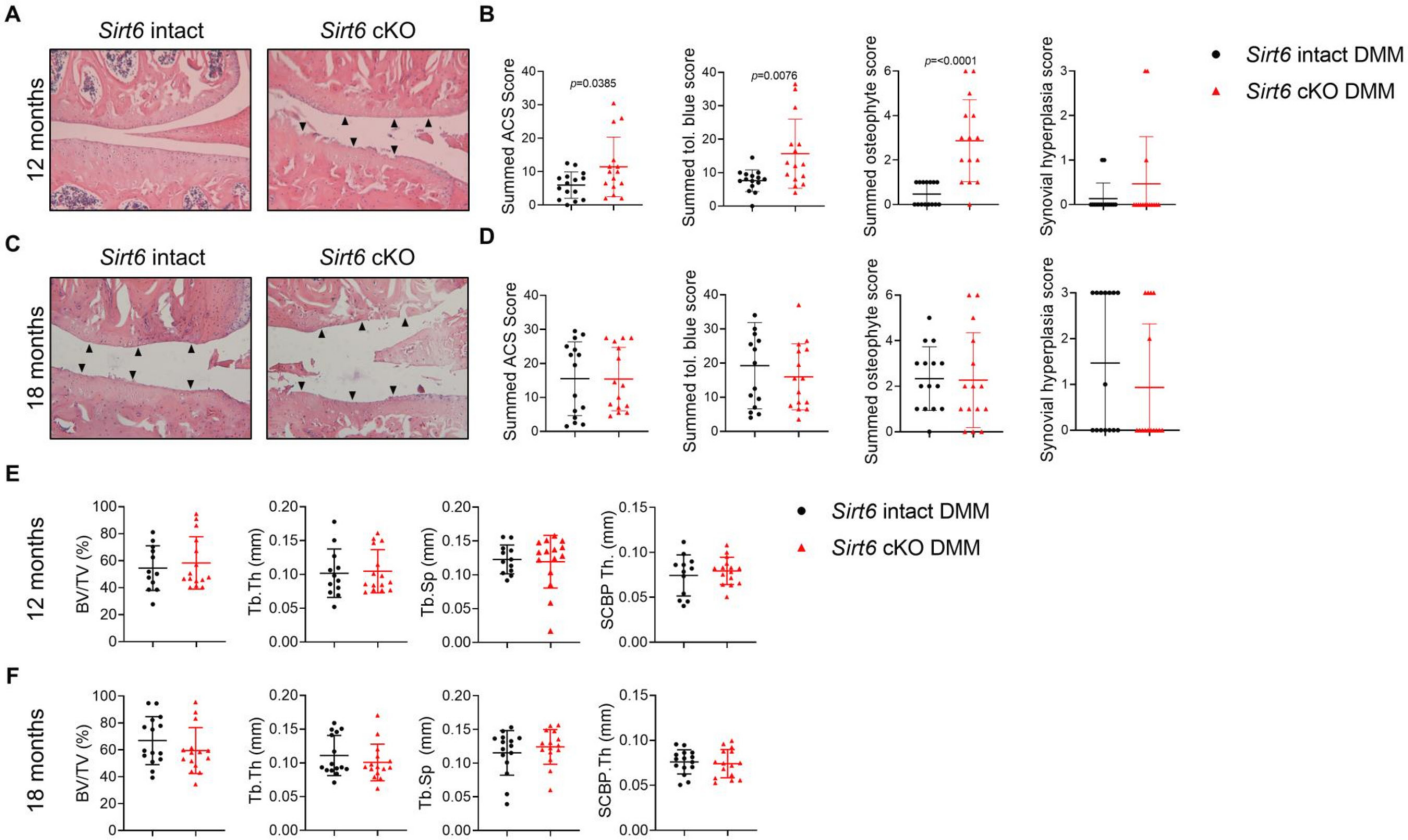
The effect of *Sirt6* deficiency on OA severity during ageing. *Sirt6* intact and *Sirt6* deficient (cKO) mice were aged 12 and 18 months and spontaneous OA was analysed by histology and micro-CT. (A,B) Representative images of H&E stained midcoronal sections showing the medial tibial plateau and medial femoral condyle from *Sirt6* intact and *Sirt6* cKO mice at 12 and 18 months of age. (C,D) Summed (MTP, MFC, LTP, LFC) ACS and toluidine blue scores, summed (MTP, LTP) osteophyte scores and synovial hyperplasia scores (medial side) at 12 and 18 months of age. Black arrows indicate areas of complete articular cartilage loss. (E) Micro-CT analysis of subchondral bone changes (BV/TV, Tb.Th, Tb.Sp, SCBP.Th) on the medial tibial plateaus of *Sirt6* intact and *Sirt6* cKO mice at 12 months and (F) 18 months of age. Individual data points are presented with mean±SD. Significant differences between groups were detected by unpaired t*-*test. Exact p values are presented. ACS, articular cartilage structure; cKO, *Sirt6*-deficient; DMM, destabilisation of the medial meniscus; LFC, lateral femoral condyle; LTP, lateral tibial plateau; MFC, medial femoral condyle; MTP, medial tibial plateau; OA, osteoarthritis; SCBP, subchondral bone plate.

To assess the effect of *Sirt6* deficiency on spontaneous, naturally occurring OA, *Sirt6* intact and *Sirt6*-deficient mice were aged 12 and 18 months with OA severity analysed as per our DMM study. At 12 months of age, *Sirt6*-deficient mice displayed a significant increase in summed ACS, toluidine blue and osteophyte scores, when compared with *Sirt6* intact controls (figure 2A,B). In agreement, detailed histomorphometric analysis showed that *Sirt6*-deficient mice displayed significant reductions in articular and calcified cartilage area on both the medial and lateral sides, when compared with *Sirt6* intact controls (tables 3 and 4). At 18 months, both genotypes displayed profound OA with loss of cartilage evident on the medial tibial plateaus, with no significant differences between genotypes (figure 2, table 3). On the lateral side, *Sirt6*-deficient mice displayed a significant decrease in articular cartilage area and thickness when compared with *Sirt6* intact controls at 18 months of age (table 4). Age or genotype had no effect on synovial hyperplasia in this ageing study (figure 2B). Similarly, we did not detect any significant differences by micro-CT on subchondral bone parameters (medial side) when analysing limbs from either genotype at both time points (figure 2E,F). The finding that synovial hyperplasia and subchondral bone changes were not affected by ageing aligns with our prior mouse studies assessing these parameters at similar time points.^17–21^ IHC to detect p16^ink4a^ as a marker of senescence was performed on mouse joint tissue sections on *Sirt6* intact and *Sirt6*-deficient mice aged 18 months. *Sirt6-*deficient mice displayed a significant increase in p16^ink4a^-positive cells in the synovium when compared with *Sirt6* intact controls (p=0.0031) (online supplemental figure 4). Taken together, these data demonstrate that cartilage-specific *Sirt6* deficiency significantly accelerates spontaneous, age-associated OA in mice.

**Table 3:** Histomorphometric analysis of *Sirt6* intact and *Sirt6* deficient (*Sirt6* cKO) mice at 12 and 18 months of age (medial tibial plateau)

Parameter	12 months			18 months		
	Sirt6 intact Mean (SD)	Sirt6 cKO Mean (SD)	Sirt6 intact versus Sirt6 cKO P value	Sirt6 intact Mean (SD)	Sirt6 cKO Mean (SD)	Sirt6 intact versus Sirt6 cKO P value
Art. Cart. area (µm^2^)	47 347 (11 461)	29 267 (15 049)	**0.0009**	32 247 (22 148)	25 679 (17 330)	0.3734
Art. Cart. thickness (µm)	36.80 (9.68)	25.12 (13.53)	**0.0112**	22.80 (17.34)	22.84 (14.68)	0.9943
Calc. Cart. area (µm^2^)	47 893 (13 631)	31 315 (13 509)	**0.0024**	38 813 (12 620)	30 779 (14 969)	0.1232
Calc. Cart. thickness (µm)	37.14 (7.41)	29.06 (10.8)	**0.0418**	34.99 (10.66)	30.46 (14.34)	0.3354
SCBP area (µm^2^)	84 978 (30 744)	121 943 (46 803)	**0.0163**	101 224 (55 040)	105 380 (52 073)	0.8333
SCBP thickness (µm)	70.53 (24.42)	90.82 (36.63)	0.0851	83.78 (34.08)	74.15 (25.48)	0.3882

Histomorphometry measurements of Art. Cart. thickness and area, Calc. Cart. thickness and area and SCBP thickness and area were analysed from midcoronal sections of mouse limbs (medial tibial plateau) from *Sirt6* intact and cKO mice at 12 and 18 months of age (n=15). Results are presented as mean±SD. Significant differences between groups were detected by Mann-Whitney U test comparing sham and DMM groups for each genotype. Exact p values are presented.

Significance values are in bold.

Art. Cart., articular cartilage; Calc. Cart., calcified cartilage; cKO, *Sirt6*-deficient; DMM, destabilisation of the medial meniscus; SCBP, subchondral bone plate.

**Table 4:** Histomorphometric analysis of *Sirt6* intact and *Sirt6* deficient (*Sirt6* cKO) mice at 12 and 18 months of age (lateral tibial plateau)

Parameter	12 months			18 months		
	Sirt6 intact Mean (SD)	Sirt6 cKO Mean (SD)	Sirt6 intact versus Sirt6 cKO P value	Sirt6 intact Mean (SD)	Sirt6 cKO Mean (SD)	Sirt6 intact versus Sirt6 cKO P value
Art. Cart. area (µm^2^)	47 487 (14 995)	37 086 (7 835)	**0.0243**	55 353 (19 226)	31 291 (12 683)	**0.0004**
Art. Cart. thickness (µm)	35.41 (10.69)	31.33 (5.88)	0.2671	44.14 (26.26)	26.26 (10.5)	**<0.0001**
Calc. Cart. area (µm^2^)	31 315 (13 509)	42 085 (9992)	**0.0279**	40 181 (9089)	34 257 (8501)	0.0759
Calc. Cart. thickness (µm)	33.96 (7.33)	27.94 (7.63)	**0.0363**	32.45 (5.15)	28.39 (5.95)	0.0554
SCBP area (µm^2^)	66 076 (22 920)	69 915 (18 988)	0.6213	68 257 (38 968)	64 297 (14 328)	0.7146
SCBP thickness (µm)	49.71 (17.84)	52.22 (15.56)	0.6854	44.95 (14.05)	44.54 (10.14)	0.9279

Histomorphometry measurements of Art. Cart. thickness and area, Calc. Cart. thickness and area and SCBP thickness and area were analysed from midcoronal sections of mouse limbs (lateral tibial plateau) from *Sirt6* intact and cKO mice at 12 and 18 months of age (n=15). Results are presented as mean±SD. Significant differences between groups were detected by Mann-Whitney U test comparing sham and DMM groups for each genotype. Exact p values are presented.

Significance values are in bold.

Art. Cart., articular cartilage; Calc. Cart., calcified cartilage; cKO, *Sirt6*-deficient; DMM, destabilisation of the medial meniscus; SCBP, subchondral bone plate.

Of interest, when analysing control mouse limbs from the 6-month-old DMM cohort (10 weeks postsham surgery) and comparing them with *Sirt6* intact controls aged 12 and 18 months, we observed an increase in BV/TV and Tb.Th values, and a reduction in Tb.Sp values in older mouse limbs. This finding suggests that ageing alone in mice increases subchondral bone sclerosis, which, to the best of our knowledge is an original finding (online supplemental figure 5).

### *Sirt6* deficiency is associated with downregulation of ECM and growth factor genes in human chondrocytes

**Figure 3:**
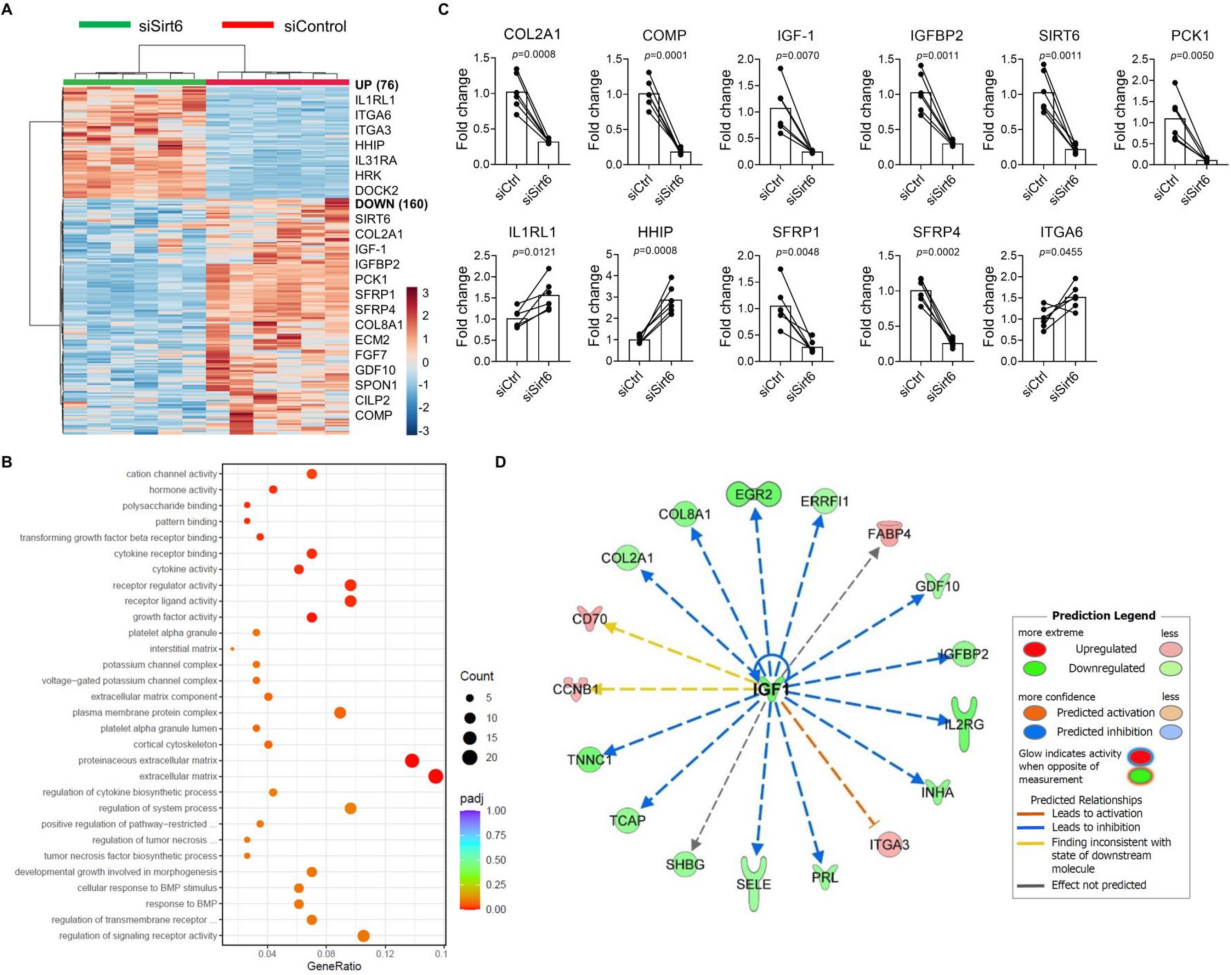
RNA-sequencing analysis of *Sirt6* depleted human chondrocytes. To assess *SIRT6*-mediated transcriptional regulation, RNA-sequencing was conducted on primary human chondrocytes nucleofected (72 hours) with small interfering RNA (siRNA) to *Sirt6* (siSirt6, *Sirt6* knockdown) and compared with cells nucleofected with a scrambled siRNA as a control (siCtrl). (A) Heatmap showing significant differentially expressed genes identified in our dataset. Selected upregulated and downregulated genes are highlighted when comparing *Sirt6* depleted cells with controls. (B) Gene Ontology (GO) enrichment showing downregulated processes in *Sirt6* depleted cells, when compared with control, are presented. (C) RT-PCR was conducted on human chondrocytes to validate expression of selected genes found in the RNA-sequencing dataset. Significant differences between groups were detected by paired t*-*test. Exact p values are presented. (D) Upstream regulator analysis using the ingenuity pathway analysis tool showing the effect of downregulated insulin-like growth factor-1 (*IGF-1*) expression on its targets.

To define the effect of *SIRT6*-mediated transcriptional regulation, RNA-sequencing was conducted on primary human chondrocytes depleted of *SIRT6* and compared with cells nucleofected with a scrambled small interfering RNA (siRNA) control (72 hours). Initial studies were undertaken to confirm *SIRT6* depletion and demonstrated that nucleofection of *SIRT6* siRNA led to a significant reduction in SIRT6 protein levels when compared with control (p=<0.0001) (online supplemental figure 6A). In our RNA-sequencing dataset, principal component analysis demonstrated that groups were strongly clustered by treatment (online supplemental figure 6B). Comparison of *SIRT6* depleted samples with controls revealed that 236 genes were differentially expressed, with 160 genes being downregulated and 76 genes being upregulated (figure 3A). Ingenuity pathway analysis demonstrated that depletion of chondrocyte *SIRT6* was predicted to increase joint inflammation, cartilage damage and the OA disease process (online supplemental figures 6C, 7). Analysis of Gene Ontology enrichment revealed that ECM, proteinaceous ECM (both cellular component) and growth factor activity (molecular function) terms were the top three repressed processes in human chondrocytes depleted of *SIRT6* when compared with controls (figure 3B).

Interrogation of differentially expressed ECM genes revealed that *COL2A1*, the primary collagen found in articular cartilage, was significantly reduced in *SIRT6* depleted cells along with other ECM genes such as *COMP, ECM2, CILP2* and *COL8A1*. In agreement, *COL2A1* (p=0.0008) and *COMP* (p=0.0001) gene expression were significantly reduced in *SIRT6*-deficient chondrocytes, when compared with controls, as assessed by qRT-PCR (figure 3C). In the context of growth factor repression, pro-anabolic insulin-like growth factor-1 (*IGF-1*) and its binding partner, IGF binding protein-2 (*IGFBP2*) were highly downregulated in *SIRT6* depleted chondrocytes, when compared with controls, which was validated by qRT-PCR (*IGF-1*; p=0.0070, *IGFBP-2*; p=0.0011). Upstream regulator analysis demonstrated that downregulation of *IGF-1* is predicted to repress various pro-anabolic cartilage genes identified in our dataset, which included *COL2A1* and *IGFBP2* (figure 3D). Other notable downregulated genes in the RNA-sequencing dataset that were validated by qRT-PCR included *SIRT6* (p=0.0011), the Wnt inhibitors, *SFRP1* (p=0.0048) and *SFRP4* (p=0.0002) and the well-described SIRT6-regulated metabolic and longevity regulator, *PCK1* (p=0.005) (figure 3C). Conversely, deficiency of *SIRT6* led to a significant increase in pro-catabolic *IL1RL1* and *HHIP* genes, which, when enhanced, have been implicated in the progression and development of OA.^22 23^ These effects were also validated by qRT-PCR (*IL1RL1*; p=0.0121, *HHIP*; p=0.0008) (figure 3A and C).

Taken together, these results demonstrate that loss of *SIRT6* significantly decreases *IGF-1* gene expression as well as a host of ECM matrix genes including *COL2A1* and promotes the gene expression of pro-catabolic genes associated with OA. As IGF-1 signalling plays a critical role in maintenance of the cartilage ECM as well as chondrocyte survival, these results stimulated us to explore the SIRT6/IGF-1 axis in chondrocytes further.

### SIRT6 regulates IGF-1 signalling in human chondrocytes

**Figure 4:**
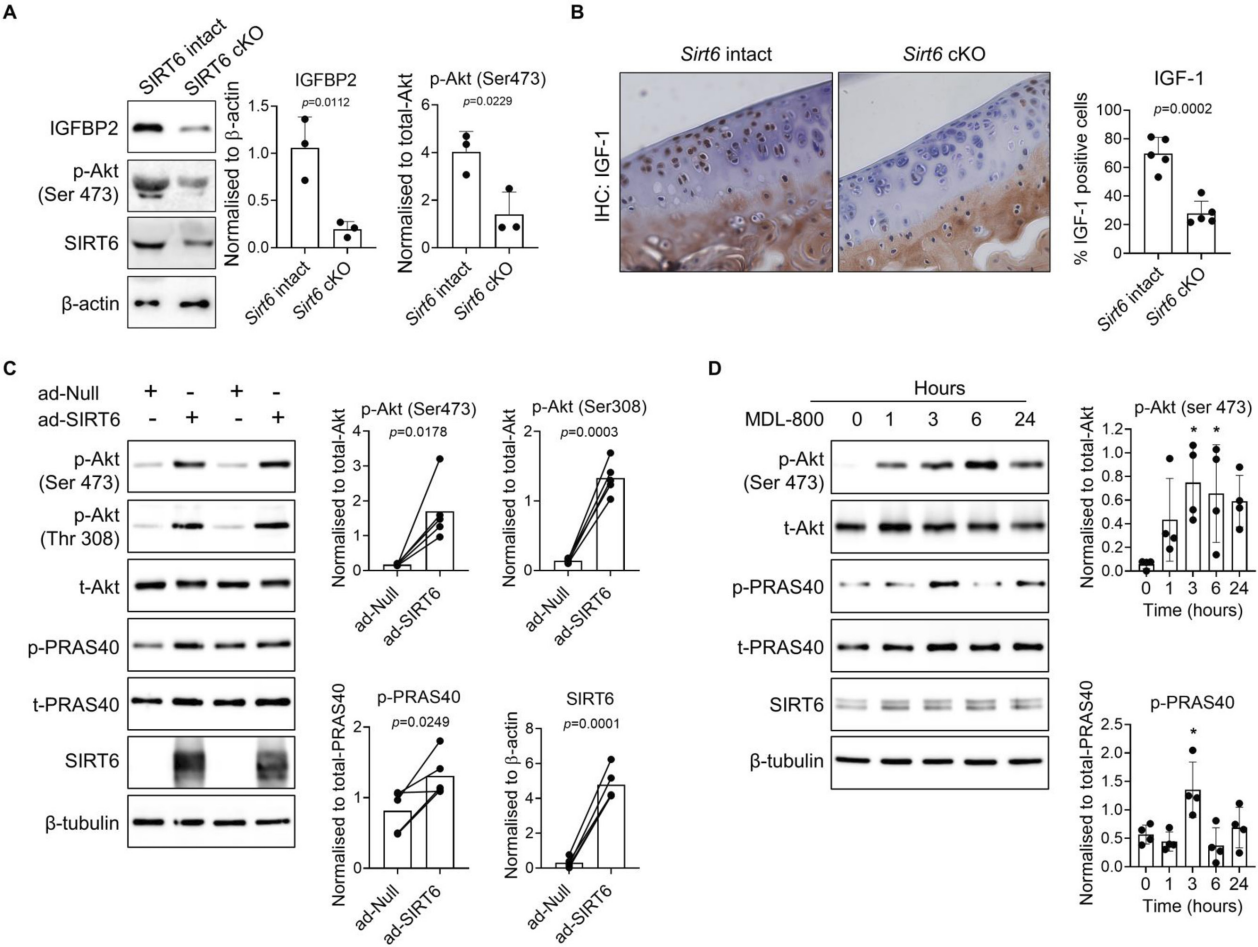
SIRT6 regulates insulin-like growth factor-1 (IGF-1) signalling in chondrocytes. (A) Femoral caps from *Sirt6* intact and *Sirt6-*deficient mice were treated with 4-hydroxytamoxifen to activate Cre-mediated recombination ex vivo. Protein levels of IGFBP2, phospho-Akt (Ser473) and SIRT6 were assessed by immunoblotting (n=3). (B) Immunohistochemistry to detect IGF-1 levels was performed on *Sirt6* intact and *Sirt6*-deficient mouse joint tissue sections derived from sham control mice from our destabilisation of the medial meniscus (DMM) study (n=5), and percentage IGF-1 positively stained cells were quantified. (C) Primary non-osteoarthritis (non-OA) older human chondrocytes were transduced with an adenoviral vector to overexpress SIRT6 or an empty vector control for 24 hours prior to immunoblotting for phospho-Akt (Ser473, Thr308), phospho-PRAS40 and SIRT6 (n=5). (D) Primary non-OA human chondrocytes were treated with MDL-800 (12.5 µM) for 0–24 hours prior to immunoblotting for phospho-Akt (Ser473), phospho-PRAS40 and SIRT6 (n=4). Presented immunoblots are representative and protein bands were normalised to total protein or housekeeping proteins as indicated. Individual data points are presented with mean±SD. Significant differences were detected by t*-*test (A–C) or two-way analysis of variance (D). *P<0.05.

To assess the effect of *Sirt6* deficiency on IGF-1 signalling in our mouse model, femoral cap cartilage derived from *Sirt6* intact and *Sirt6*-deficient mice was dissected and cultured as explant cultures ex vivo. Explants were treated with 4-hydroxytamoxifen daily for 96 hours to induce Cre-mediated depletion of *Sirt6* and then chondrocytes were extracted and processed for immunoblotting to detect IGFBP2, and phosphorylated Akt, as a marker of IGF-1 signal pathway activation. *Sirt6* depleted explants displayed a significant reduction in basal IGFBP2 (p=0.0112) and phospho-Akt^ser473^ (p=0.0229) protein levels when compared with *Sirt6* intact femoral cap explants (figure 4A). IHC performed on joint tissue sections derived from *Sirt6* intact and *Sirt6*-deficient sham control mice from our DMM study demonstrated that IGF-1 levels were significantly decreased in *Sirt6*-deficient mouse cartilage when compared with *Sirt6* intact controls (p=0.0002), which aligns with our RNA-sequencing data in human chondrocytes with *Sirt6* knockdown (figure 4B).

As *Sirt6* deficiency decreased the IGF-1/Akt axis in mouse cartilage, we next aimed to test the effect of SIRT6 activation to promote IGF-1 signalling in chondrocytes. Primary human chondrocytes were transduced with an adenoviral vector encoding *SIRT6* (24 hours) to increase SIRT6 activity, or an empty vector (null) control as we have previously described.^8^ Overexpression of SIRT6 significantly increased basal phosphorylation of Akt at Ser^473^ (p=0.0178) and Thr^308^ (p=0.0003) and increased phosphorylation of proline-rich Akt substrate (PRAS40) (p=0.0249), a marker of Akt activity,^24 25^ when compared with null empty vector controls (figure 4C). Next, we treated primary human chondrocytes with the small molecule activator of SIRT6, MDL-800, that has been previously shown by others to increase SIRT6 activity up to 22-fold.^26^ We isolated chondrocyte histones and performed immunoblotting for the acetylated form of the SIRT6 substrate, H3K9 (H3K9ac), an inverse marker of SIRT6 activity.^8^ Treatment of chondrocytes with MDL-800 (12.5 µM, 24 hours) led to a significant decrease in the basal acetylated form of H3K9 (p=0.0001), indicating enhanced SIRT6 activity compared with controls (online supplemental figure 8). In total cell lysates, MDL-800-induced SIRT6 activation led to a significant increase in basal phospho-Akt^ser473^ and phospho-PRAS40 that peaked at 3 hours of treatment (p=0.0170, p=0.0120, respectively) (figure 4D). SIRT6 protein levels did not change in response to treatment with MDL-800 over the time course studied, which is in accordance with others.^26 27^ Collectively, these gain-of-function and loss-of-function studies demonstrate that SIRT6 is a critical regulator of the pro-anabolic IGF-1 signalling pathway in mouse and human chondrocytes and decreases catabolic signalling events associated with OA.

## Discussion

This study demonstrates that *Sirt6* deficiency in the cartilage significantly enhances OA severity in response to DMM surgery in younger mice and accelerates spontaneous OA in older mice, characterised by profound cartilage degradation, subchondral bone sclerosis and osteophyte formation. Mechanistically, RNA-sequencing analysis revealed that *SIRT6* depletion in chondrocytes significantly repressed *IGF-1* gene expression and a plethora of cartilage ECM-associated genes including *COL2A1*. Furthermore, qRT-PCR, IHC and immunoblotting analyses demonstrated that downregulation of SIRT6 significantly represses *IGF-1* and the IGF-1/Akt signalling pathway, whereas SIRT6 overexpression, or activation using MDL-800, significantly increases Akt activity.

Our finding that cartilage-specific *Sirt6* deficiency increased OA in mice agrees with a recently published study by Ji *et al*,^7^ which demonstrated that mouse chondrocytes depleted of *Sirt6* display upregulated catabolic IL-15/JAK/STAT signalling, leading to senescence and enhanced OA in vivo.^7^ Our finding that p16^ink4a^ levels were significantly increased in the synovium of old *Sirt6*-deficient mice, when compared with controls, aligns with this concept and suggests that cartilage damage as a result of *Sirt6* deficiency could promote the release of catabolic factors that are released into the joint to promote senescence. Alongside these lines of data, prior findings conducted in chondrocytes report that SIRT6 abrogates NF-κB signalling,^8 11^ enhances DNA repair pathways^12^ and promotes resistance to oxidative stress conditions.^8^ Along with our finding that *Sirt6* critically regulates IGF-1 signalling, these data demonstrate that SIRT6, which is positioned at the apex of many important age-related and OA-related pathways, is a master regulator of various homeostatic processes in joint tissues, as has been demonstrated in a plethora of different cell types in ageing and disease-associated contexts.^1 2^

This previously unrecognised role of *Sirt6* as a regulator of IGF-1 signalling in chondrocytes underscores its importance in overall joint tissue integrity and is a plausible mechanism for the reduction in cartilage ECM gene expression observed in human chondrocytes as well as the severe OA phenotype in our mouse model. Indeed, IGF-1 is known to be a major regulator of articular cartilage ECM integrity by stimulating the synthesis of collagens and proteoglycans.^28–30^ Furthermore, our prior work in chondrocytes demonstrates that IGF-1-mediated ECM synthesis is dependent on phosphorylation and activation of Akt.^31^ However, in older and OA chondrocytes, and in chondrocytes treated with oxidative stress inducers to simulate ageing conditions, IGF-1/Akt signalling is inhibited leading to a significant reduction in proteoglycan synthesis, activation of pro-catabolic signalling events associated with ECM catabolism such as p38 and ERK phosphorylation and chondrocyte cell death.^24 25 31–34^ Importantly, our prior work demonstrates that chronic IGF-1 deficiency increases OA severity in older rats, characterised by enhanced proteoglycan loss and cartilage damage when compared with controls.^35^

Conversely, activation of Akt is associated with restoration of homeostatic mitochondrial function, increased collagen II synthesis and repression of MMP-13 levels which led to protection against cartilage degradation in a rat post-traumatic OA model.^36 37^ As such, the discovery of novel strategies aimed at maintaining homeostatic IGF-1/Akt signalling in cartilage during ageing and/or in response to joint injury are considered critically important for supporting the chondrocyte phenotype and protecting against OA.^38^

In this study, we used adenoviral vectors to overexpress SIRT6 or the commercially available small molecule activator of SIRT6, MDL-800, as tools to enhance SIRT6 activity in human chondrocytes and analysed Akt signalling. Activation of SIRT6 by both methods led to a robust increase in phosphorylation of Akt and its downstream marker of activity, PRAS40. To our knowledge, this is the first demonstration of SIRT6-mediated activation of Akt in chondrocytes. Furthermore, this finding builds on our prior work showing that SIRT6 overexpression is beneficial to chondrocytes by upregulating antioxidant levels as well as promoting detoxification of oxidative stress levels of H_2_O_2_ that are associated with ageing and OA.^8^ MDL-800 has recently been shown to upregulate *COL2A1* and *ACAN* gene expression in chondrocytes.^7^ In addition, MDL-800 can protect against DMM-induced cartilage damage when administered into the joints of young mice.^7^ These lines of evidence suggest that MDL-800-mediated activation of SIRT6 may represent a novel and viable therapeutic candidate for OA.

Previous reports have detailed the effect of sirtuins to directly regulate IGF-1/Akt signalling events in several tissues. For example, SIRT1, which is the most studied mammalian sirtuin isoform, mediates deacetylation of Akt to enhance the binding of Akt and PDK1 to PIP3 to enhance IGF-1 signalling in HEK293T cells.^39^ Furthermore, activation of SIRT1 by several compounds has been shown to attenuate lipopolysaccharide and IL-1β-induced inhibition of Akt phosphorylation to repress inflammatory mediators, enhance cell survival and promote the synthesis of ECM components such as aggrecan, collagen II and SOX9 in nucleus pulposus cells.^40 41^ Although data demonstrating a direct role for SIRT6 to activate IGF-1 signalling are sparse, recent evidence in tumour cell lines demonstrates that overexpression of SIRT6 can deacetylate and phosphorylate Akt to enhance activation of the downstream apoptosis inhibitor protein, XIAP, and promote cell survival.^42^ These lines of evidence agree with our findings and suggest that SIRT6 may physically interact and deacetylate members of the IGF-1-pathway to promote activation. Elucidating the epigenetic and post-transcriptional effects of SIRT6 on the IGF-1 pathway requires further study.

The observation that IGFBP2 was repressed in *SIRT6* depleted human chondrocytes and mouse cartilage provides important insights into IGF-1 regulation in chondrocytes. Although increased IGFBP levels have been shown to block IGF-1 binding to the IGF receptor,^43^ several gain-of-function and loss-of-function studies have demonstrated that enhanced IGFBP2 levels increases the phosphorylation and activation of Akt in musculoskeletal tissues,^44 45^ which aligns with our findings. Our additional finding that the Wnt antagonists, *SFRP1* and *SFRP4*, were downregulated in *SIRT6* depleted cells suggests that loss of *SIRT6* may lead to aberrant activation of Wnt signalling that could contribute to exacerbation of inflammatory pathways associated with OA, as has been well described in chondrocytes.^46^ Conversely, *IL1RL1* and *HHIP* gene expression were found to be significantly elevated in *SIRT6* depleted chondrocytes in our study, relative to controls. Upregulation of these genes have been observed in OA tissues and are associated with increased joint inflammation, cytokine release and OA severity in mouse models of OA.^22 47^ Taken together, these lines of evidence add to the hypothesis that loss of *Sirt6* causes a pathological imbalance between pro-anabolic and pro-catabolic signalling events that lead to cartilage degradation and OA. Whether SIRT6 directly, or indirectly regulates these newly identified SIRT6 targets, and in particular IGF-1 and downstream IGF-1 targets, was beyond the scope of the current study, but is a focus of future investigation in cartilage as well as all other joint tissues affected by OA.

There were some limitations in this study. We only used male mice since they are more susceptible to developing OA^48^ and our study design required a large number of mice. Future studies would be needed to examine for differences in female mice. We also did not include pain measures, although with the extent of the histological OA noted in the *Sirt6* knockouts we would expect some degree of pain-related behaviour. Our ongoing research aims to further define the SIRT6-associated mechanisms that drive OA development as well as the potential therapeutic effect of commercially available SIRT6 activators, such as MDL-800 or UBCS039,^49^ to protect against OA in other joint tissue cells and multiple animal models of OA in both sexes.

In conclusion, the current study was motivated by several reports, including our own, showing that SIRT6 activity^8^ or SIRT6 levels^11^ are reduced with age and OA in chondrocytes. We demonstrate here that cartilage-specific depletion of *Sirt6* increases post-traumatic OA in younger mice and accelerates age-associated OA in older mice. Furthermore, we identify the pro-anabolic IGF-1 pathway as a major target of SIRT6 in chondrocytes. *SIRT6* depletion significantly repressed *IGF-1* levels which is a potential mechanism responsible for the observed reduction of cartilage ECM genes in the absence of *SIRT6*. The development and progression of OA is multifactorial and multiple homeostatic anabolic and pro-catabolic pathways are altered leading to damage to all joint tissue structures. Recent in vitro and in vivo studies demonstrate that SIRT6 can regulate an array of biological processes implicated in ageing, healthspan as well as regeneration,^1 4 5 14 50^ which may hold great promise for therapies aimed at slowing, stopping or reversing the OA process. Thus, targeted strategies that maintain or activate SIRT6 to promote the chondrocyte phenotype and maintain cartilage ECM integrity represent promising avenues for both post-traumatic and age-associated OA therapy as well as other diseases of the joint.

## Data Availability

Data are available on reasonable request. RNA-sequencing dat are available at GEO accession GSE235082.
