# Supplementary material for: Cartilage-specific Sirt6 deficiency represses IGF-1 and enhances osteoarthritis severity in mice
Source: Ann Rheum Dis. 2023 Aug 7;82(11):1464–73. doi: 10.1136/ard-2023-224385 (PMC10579179; doi:10.1136/ard-2023-224385)
Supplement: Supplementary data [file ard-2023-224385supp001.pdf]

Online Supplementary Materials

Cartilage-specific *Sirt6* deficiency represses IGF-1 and enhances osteoarthritis severity in mice.

Authors: John A. Collins, Cheeho Kim, Ashley Coleman, Abreah Little, Matheus Moreira Perez, Emily-Jane Clarke, Brian O. Diekman, Mandy J. Peffers, Susan Chubinskaya, Ryan E. Tomlinson, Theresa A. Freeman, Richard F. Loeser.

Supplementary Materials and Methods

Antibodies and reagents. Antibodies to SIRT6 (12486), IGFBP2 (3922), phospho-Akt (Ser473) (4060), phospho-Akt (Thr308) (4056), total-Akt (9272), phospho-PRAS40 (2997), total PRAS40 (2610), Histone 3 (9715), β-actin (4967), and β-tubulin (2146) were purchased from Cell Signaling Technologies. Antibodies to IGF-1 and p16^{ink4a} (IHC) were purchased from Abcam (ab9572 and ab211542) and the SIRT6 antibody (IHC) was purchased from LS-Bio (LS-B5589). The antibody to H3K9ac (06-942), 4-hydroxytamoxifen (H7904), and MDL-800 (SML2529) were purchased from Millipore-Sigma.

Mouse Studies. Animal studies were approved by the University of North Carolina and Thomas Jefferson University Institutional Animal Care and Use Committees following guidelines from the National Institutes of Health Guide for the Care and Use of Laboratory Animals. Mice were housed with an average of 4 mice per cage and had access to *ad libitum* water and food. All studies were performed on male mice (C57Bl/6Jx129SxFVB/NJ background) due to prior studies demonstrating an increase in OA severity in male mice given DMM surgery, when compared to female mice (1). *Sirt6*$^{fl/fl}$ mice were obtained from Jackson laboratories (stock #017334, Bar Harbor, ME, USA). *Aggrecan-Cre*^{ERT2} mice were kindly provided by Dr. Benoit de Crombrugghe (University of Texas M.D. Anderson Cancer Center) and are now available from Jackson Laboratories (stock #019148). *Sirt6*$^{fl/fl}$ mice were crossed with *Aggrecan-Cre*^{ERT2} mice to obtain *Sirt6*$^{fl/fl}$;*Aggrecan-Cre*^{ERT2} mice (*Sirt6* deficient mice, *Sirt6* cKO). Littermate *Sirt6*$^{fl/fl}$ mice (in the absence of *Aggrecan-Cre*^{ERT2}) were used as *Sirt6* intact controls.

Collins JA, *et al. Ann Rheum Dis* 2023; 82:1464–1473. doi: 10.1136/ard-2023-224385

Genotyping was carried out using PCR protocols provided by Jackson Laboratories. At 12 weeks of age, mice received daily intraperitoneal injections of tamoxifen (40 mg/kg diluted to 10 mg/ml in corn oil) for 5 days to activate cre recombinase activity of *Aggrecan-Cre^{ERT2}* and induce *Sirt6* deficiency. To control for the effects of tamoxifen, *Sirt6* intact mice (without *Aggrecan-Cre^{ERT2}*) also received tamoxifen dosing. Our prior data demonstrates that treatment with tamoxifen alone or the *Aggrecan-Cre^{ERT2}* allele alone has no effect on OA outcomes in the DMM model (2). Deletion of *Sirt6* in this model has been previously established *ex vivo* by our group (3) and was confirmed in the present study using IHC and an antibody to SIRT6 on mouse joint tissue sections (Supplementary Figure 1).

To assess the effect of *Sirt6* deficiency on surgery-induced OA, mice from each genotype (*Sirt6* intact or *Sirt6* cKO*)* were randomized to surgical groups and DMM or sham surgery was performed on the right knees of mice at 16 weeks of age, as we (2, 4) and others (1, 5) have described. Mice were sacrificed at 6- or 10-weeks after surgery as these are time points that have been shown to elicit moderate and moderate-severe OA (6, 7). We used *n*=13-15 mice for DMM groups and an *n*=6-9 for sham control groups. To assess the effects of *Sirt6* deficiency on age-associated OA, male *Sirt6* intact and *Sirt6* cKO mice received injections of tamoxifen at 12 weeks of age and were then aged to 12 or 18 months (*n*=15 per age group and genotype). Mice assigned to the 18-month-old groups received a second round of tamoxifen dosing (daily for 3 days) at 12 months of age. Mouse numbers were chosen based on power analyses from our previously published studies (2, 4). We did not observe significant changes in body weights or gross morphological differences between mice in our study.

Micro computed tomography (µCT) analysis. Mice were sacrificed and hindlimbs were harvested, surrounding muscles were removed, and hindlimbs were fixed in 10% formalin (48 hours) prior to µCT analysis using a Bruker SkyScan 1275 scanner with 7µm^3 voxel size and X-ray energies of 70 kVp and 142 µA, as previously described (8). Scans comprised of approximately 1300 slices spanning the femur, tibia, and knee joint. µCT reconstructions and quantitative analyses were performed using the SkyScan CT Analyzer (CTan) from regions of interest (medial tibia) that spanned approximately 160 coronal slices. The following structural parameters were analyzed; tibial subchondral bone volume fraction (BV/TV), Trabecular Thickness (Tb.Th), Trabecular Separation (Tb.Sp), Subchondral Bone Plate thickness (SCBP),

osteophyte volume, and max. osteophyte area. Osteophytes were observed as outgrowing bone protrusions compared with sham limbs, as described in (9).

Histological analysis of OA. After µCT analysis, hindlimbs were decalcified (EDTA, 19%, 21 days), processed, embedded in paraffin, and sectioned (5 µm thickness) along the coronal plane, as described previously (10-13). Approximately 12 midcoronal sections were collected per limb (surgical limb only) for histological and histomorphometric analysis. Mid-coronal sections were stained with hematoxylin and eosin (H&E) or toluidine blue. Articular Cartilage Structure (ACS) score (0-12 scale) and toluidine blue score (0–12 scale) were analyzed on medial tibial plateaus (MTP), lateral tibial plateaus (LTP), medial femoral condyles (MFC), and lateral femoral condyles (LFC), as previously described (13). Osteophytes (0–3 scale) were scored on the MTP and LTP and synovial hyperplasia (0–3 scale) was analyzed in the medial compartment (13) from H&E stained sections. All sections were scored by two individuals blinded to experimental groups who had been trained to use the grading schemes. Histomorphometric measurements of articular cartilage thickness and area, calcified cartilage thickness and area, and subchondral bone thickness and area were analyzed on the MTP and LTP, as previously described (12, 13), using ImageJ software.

Cell culture of human chondrocytes and mouse femoral cap explants. Normal (non-osteoarthritic) human primary human articular chondrocytes were isolated from tali cartilage of deceased human tissue donors provided by the National Disease Research Interchange and a collaboration between The Gift of Hope Organ and Tissue Donor Network (Itasca, IL) and Rush University Medical Center (Chicago, IL), with IRB approval. Cartilage was macroscopically inspected prior to dissection and scored for degeneration using a modified version of the 5-point Collins grading system (14). Only normal appearing cartilage was used (grade 0-2). Normal cartilage was obtained from a total of 23 donors (male, 17; female, 6; age range 39–84 years, av. age; 60.3±12.6 years). Chondrocytes were isolated by sequential digestion of cartilage with Pronase and Collagenase P and maintained in monolayer culture in Dulbecco's Modified Eagle Medium (DMEM) supplemented with 10% Fetal Bovine Serum (FBS) and antibiotics. Chondrocytes (passage 0) were cultured at high density and grown to experimental confluence (80-90%) and cultured in serum-free conditions prior to experimental incubations.

Collins JA, *et al. Ann Rheum Dis* 2023; 82:1464–1473. doi: 10.1136/ard-2023-224385

Femoral head cartilage from the hip joints of 8-week-old *Sirt6* intact and *Sirt6* cKO mice (n=3) were dissected and cultured as explants as we have previously described (3, 15). Femoral cap explants were treated with 5 µM 4-Hydroxytamoxifen daily for 4 days to activate *Cre* recombinase activity of *Aggrecan-Cre*^{ERT2} and induce *Sirt6* deficiency. Cartilage explants were maintained in DMEM supplemented with 10% FBS and antibiotics.

Knockdown of SIRT6 by Amaxa nucleofection of siRNA. Knockdown of SIRT6 was achieved by electroporation of SIRT6 siRNA (SignalChem) using Amaxa Nucleofection (Lonza). Human chondrocytes were cultured in monolayer in 10 cm dishes until 80% confluency was achieved. Chondrocytes were then isolated by incubation in a Collagenase P and Pronase solution (1mg/ml in serum containing media) until chondrocytes were in a single cell suspension (60-120 mins). Cells were centrifuged (90 xg, 8 mins) and washed twice in 1X PBS. Chondrocytes were pelleted and resuspended in Amaxa P3 Primary Cell 4-D Nucleofector Reagent (100 µl/ml) (Lonza) containing 2 µM SIRT6 siRNA or a scrambled control and entered into Amaxa P3 Primary Cell 4-D Nucleocuvette vessels (Lonza). Electroporation was carried out in a 4-D Nucleofector (Lonza) (program ER-100). Nucleofected cell suspensions were incubated at RT for 10 mins prior to incubation in 20% serum containing media for 24 hours (37°C, 5% CO_2). Media was aspirated after 24 hours and replaced with 10% serum containing media for 48 hours prior to immunoblot analysis.

SIRT6 overexpression and activation studies. For SIRT6 overexpression studies, human chondrocytes were incubated in serum-free media containing 1M CaCl (25µl/ml) and an adenoviral vector encoding SIRT6 (ad-SIRT6) or an empty vector control (ad-Null) (both $4x10^8$ viral particles/ml) (25µl/ml) for 2 hours (37°C) as we have previously described (3, 15, 16). Chondrocytes were then maintained in serum containing DMEM for 48 hours prior to lysis. For SIRT6 activation studies, confluent human chondrocytes were treated with the small molecule activator of SIRT6, MDL-800 (12.5 µM), for 0-24 hours and then total cell lysates were prepared. SIRT6 activity was analyzed in human chondrocyte lysates by isolation of histones and analysis of H3K9 acetylation (H3K9ac) as we have previously described (3). Analysis of H3K9ac is routinely performed by our lab, and others, as an inverse marker of SIRT6 activity (3, 17-22). Histones were isolated from total cell lysates using the Histone Extraction Kit (Abcam).

RNA-sequencing analysis.

Total RNA Extraction, Library Preparation and RNA-sequencing

SIRT6 was depleted in human chondrocytes by Amaxa nucleofection of siRNA. Chondrocytes nucleofected with a scrambled siRNA were used as controls as described above. Total RNA from cells was extracted using the NucleoSpin RNA mini kit for RNA purification (Macherey-Nagel) according to manufacturer's instructions. Library preparation, RNA-sequencing, and initial data analysis was carried out by Novogene. In brief, RNA quality was assessed using an Agilent 2100 bioanalyzer. Messenger RNA was purified from total RNA using poly-T oligo-attached magnetic beads. Following fragmentation, the first strand cDNA was synthesized using random hexamer primers. Second strand cDNA synthesis was undertaken using either dUTP for directional library or dTTP for non-directional library. For non-stranded library preparation polyA enrichment was utilized to capture mRNA fragments. The library was checked with Qubit and real-time PCR for quantification and bioanalyzer for size distribution detection. Quantified resulting pooled libraries were sequenced on Novaseq 6000 (Illumina) with 270-300bp paired-end reads. RNA-sequencing data has been submitted to the National Centre for Biotechnology Information Gene Expression Omnibus (NCBI GEO) under Array Express accession number GSE235082.

RNA-sequencing Data Analysis

Overall quality of the next-generation sequencing data was evaluated using in-house perl scripts. Clean reads were obtained following removal of reads containing adapters, reads containing ploy-N and low-quality reads from raw data. All the downstream analyses were based on the clean data with high quality. Reads were mapped against the reference genome Homo Sapiens (GRCh38/hg38) and gene model annotation files were downloaded from genome website directly (http://ftp.ensembl.org/pub/). Index of the reference genome was built using Hisat2 v2.0.5 and paired-end clean 2 reads were aligned to the reference genome using Hisat2 v2.0.5. FeatureCounts v1.5.0-p3 was used to count the reads numbers mapped to each gene(23-25). Parameters for mismatches were 2-6 max and min penalties for mismatch; lower qual = lower penalty <2,6>. of each gene was calculated based on the length of the gene and reads count mapped to this gene. Differential expression analysis of two conditions/groups (two biological

replicates per condition) was performed using the DESeq2 R package (1.20.0)(26). The resulting P-values were adjusted using the Benjamini and Hochberg's approach to control the false discovery rate. Genes with an adjusted P-value <=0.05 found by DESeq2 were assigned as differentially expressed. Prior to differential gene expression analysis, for each sequenced library, the read counts were adjusted by edgeR program package through one scaling normalized factor. Differential expression analysis of two conditions was performed using the edgeR R package (3.22.5). Corrected P-value of 0.05 and absolute fold change of 2 were set as the threshold for significantly differential expression.

Metaboanalyst Analysis

RNA expression was normalized by median and pareto scaled prior to multivariate analysis. All principal component analysis (PCA) and heat map analysis was conducted using MetaboAnalyst 4.0 (http://www.metaboanalyst.ca).

Ingenuity Pathway Analysis

Ingenuity Pathway Analysis (IPA, Ingenuity Systems, Redwood City, California, US), was used to analyze the RNASeq data for canonical pathways, upstream regulators, and disease and function analysis using the list of differentially expressed genes (log2FC cutoff of ±2 and FDR<0.05). All molecules were overlaid onto a global molecular network contained in the Ingenuity Knowledge base. Networks of network-eligible molecules were algorithmically generated based on their connectivity. The functional analyses identified the canonical pathways, upstream regulators and biological functions and diseases that were most significant to the data set. A right-tailed Fisher's exact test was used to calculate the raw p-values. The z-score was used to predict the activation or inhibition state of the molecules in our datasets. Canonical pathways, upstream regulators and biological functions and diseases which were likely activated (based on the pattern of differentially abundant proteins or metabolites) were presented in orange (positive z-score), those that were likely inhibited were presented in blue (negative z-score), and those with a z-score which is zero (or close to zero) or ineligible for prediction were presented in white or grey, respectively.

RT-qPCR from human chondrocytes. RNA isolation from human chondrocytes was performed using the NucleoSpin RNA mini kit for RNA purification (Macherey-Nagel) according to

manufacturer's instructions. Reverse transcription (500ng of RNA) was performed using the RNA to cDNA EcoDry Premix (Takara) and PCR was performed using *Power* SYBR® Green PCR mastermix (Applied Biosystems) and gene-specific oligos purchased from Integrated DNA Technologies (IDT) on a QuantStudio™ 3 real-time PCR system (Applied Biosystems). Primer sequences used in the study are presented in Supplementary Table 1. Gene expression was normalized to HPRT1 as a housekeeping control and analysis of gene expression was conducted using the $2^{-\Delta\Delta Cq}$ method (27, 28). Data is presented as fold change comparing Sirt6 depleted cells to controls.

Immunoblotting. After experimental stimulations, human chondrocytes were incubated in standard lysis buffer for 30 mins under gentle agitation at 4°C. Mouse femoral cap cartilage explants were lysed and homogenized using a Precellys homogenizer (Bertin Technologies) as previously described (3). Cell lysates were centrifuged at 12,000 rpm (10 mins) to remove non-soluble proteins and protein concentrations were determined using the Pierce™ micro-BCA assay (Thermo Fisher Scientific). Immunoblotting was performed on cell lysates under reducing conditions (10% β-mercaptoethanol) using specified antibodies as we have previously described (15). Immunoblots were stripped and reprobed with total antibodies or β-tubulin/β-actin as loading controls. All antibodies were used at a 1:1000 dilution (in Tris-buffered saline with 0.1% Tween (TBST) and 5% blotting grade blocker (Bio-Rad)). Densitometric analysis was performed using ImageJ software.

Immunohistochemistry (IHC). IHC was performed on mouse joint tissue sections using the VECTASTAIN™ Elite™ ABC Kit as we have previously described (3). Joint tissue sections were incubated in 3% H_2O_2 (10 min) prior to sodium citrate antigen retrieval (10 mM sodium citrate buffer, pH 6.0) at 90ºC for 30 minutes. Sections were rinsed in PBS, blocked in 5% donkey serum, and incubated overnight at room temperature in primary antibodies to SIRT6, IGF-1, or $p16^{ink4a}$. Sections were rinsed in PBS and then incubated in biotinylated secondary antibody for 1 hour. The secondary antibody was removed and sections were incubated in VECTASTAIN™ Elite™ Avidin-Biotin Complex solution for 30 min and then incubated in Vector™ DAB peroxidase substrate. Slides were counterstained with hematoxylin and imaged using an EVOS M5000 imaging system. Percent positive cells were analyzed using ImageJ.

Collins JA, *et al. Ann Rheum Dis* 2023; 82:1464–1473. doi: 10.1136/ard-2023-224385

Statistical Analysis. Statistical analysis was performed in GraphPad Prism version 9. All data are presented as mean values ± SD and individual data points are presented in graphs. Exact biological replicates are presented in figure legends. Normal distribution of data was tested using the D'Agostino and Pearson normality test and normally distributed data was analyzed using paired or unpaired students t-tests when comparing two groups, or one-way ANOVA as indicated in figure legends. Data that was not normally distributed was analyzed by Mann-Whitney tests comparing experimental groups to controls. Significant *p*-values are presented in graphs with a *p*-value of <0.05 deemed significant.

Collins JA, *et al. Ann Rheum Dis* 2023; 82:1464–1473. doi: 10.1136/ard-2023-224385

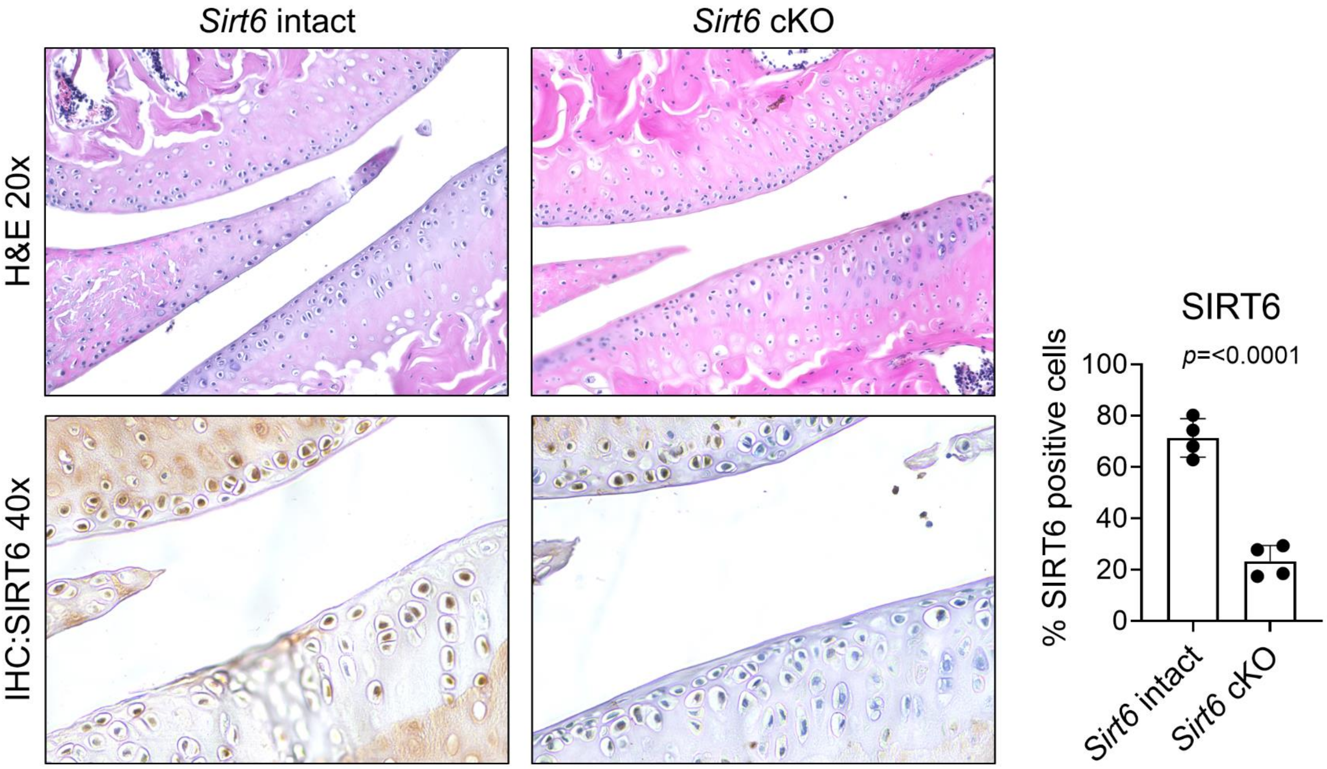

Supplementary Figure 1. SIRT6 levels are decreased in chondrocytes from *Sirt6$^{fl/fl}$;Aggrecan-Cre^{ERT2}* mice. To demonstrate SIRT6 deficiency *in vivo*, SIRT6 levels in chondrocytes from *Sirt6$^{fl/fl}$* (*Sirt6* intact) and *Sirt6$^{fl/fl}$;Aggrecan-Cre^{ERT2}* (*Sirt6* cKO) contralateral sham mouse limbs were analyzed by immunohistochemistry. H&E stained midcoronal sections (upper panels) showing undamaged cartilage from the lateral medial femoral condyle and tibial plateaus of *Sirt6* intact and *Sirt6* cKO mice (n=4). Immunohistochemistry to detect SIRT6 levels (lower panels) was performed on midcoronal sections from the same mouse joints (n=4)

and percentage SIRT6 positively stained cells were quantified. Individual data points are presented with mean ± standard deviation (SD). Significant differences between groups were detected by unpaired *t*-test. Exact *p*-values are presented.

Collins JA, *et al. Ann Rheum Dis* 2023; 82:1464–1473. doi: 10.1136/ard-2023-224385

Gene	Sequence (5' – 3')	Amplicon (bp)
hCOMP	F: CCCCAATGAAAAGGACAACTGC<br>R: GTCCTTTTGGTCGTCGTTCTTC	121
hCOL2A1	F: AGAACTGGTGGAGCAGCAAGA<br>R: AGCAGGCGTAGGAAGGTCAT	142
hHHIP	F: TCTCAAAGCCTGTTCCACTCA<br>R: GCCTCGGCAAGTGTAAAAGAA	108
hHPRT1	F: ATGGACAGGACTGAACGTCTTGCT<br>R: TTGAGCACACAGAGGGCTACAATG	80
hIGF1	F: GAGTCTGGCCAAAACGGTAA<br>R: CCCAAATGGATGGTGTTTTC	102
hIGFBP2	F: ACAACCTCAAACAGTGCAAGA<br>R: GGCTCCCTGGATCAGCTTC	92
hIL1RL1	F - GAAAACCTAGTTACACCGTGGAT<br>R - GCAAACACACGATTTCTTTCCTG	82
hITGA6	F: ATGCACGCGGATCGAGTTT<br>R: TTCCTGCTTCGTATTAACATGCT	160
hPCK1	F - GAGAAAGCGTTCAATGCCAG<br>R - ATGCCGATCTTTGACAGAGG	107
hSIRT6	F - GCCTGGTCATCGTCAACCTG<br>R - TCATGACCTCGTCAACGTAGC	84
hSFRP1	F - TGAGGCCATCATTGAACATC<br>R - TCATCCTCAGTGCAAACTCG	50

hSFRP4	F - CTGCCCCATCAAGATGTTCT	53
	R - ATCATCCTTGAACGCCACTC	

Supplementary Table 1. Human primers used in the study.

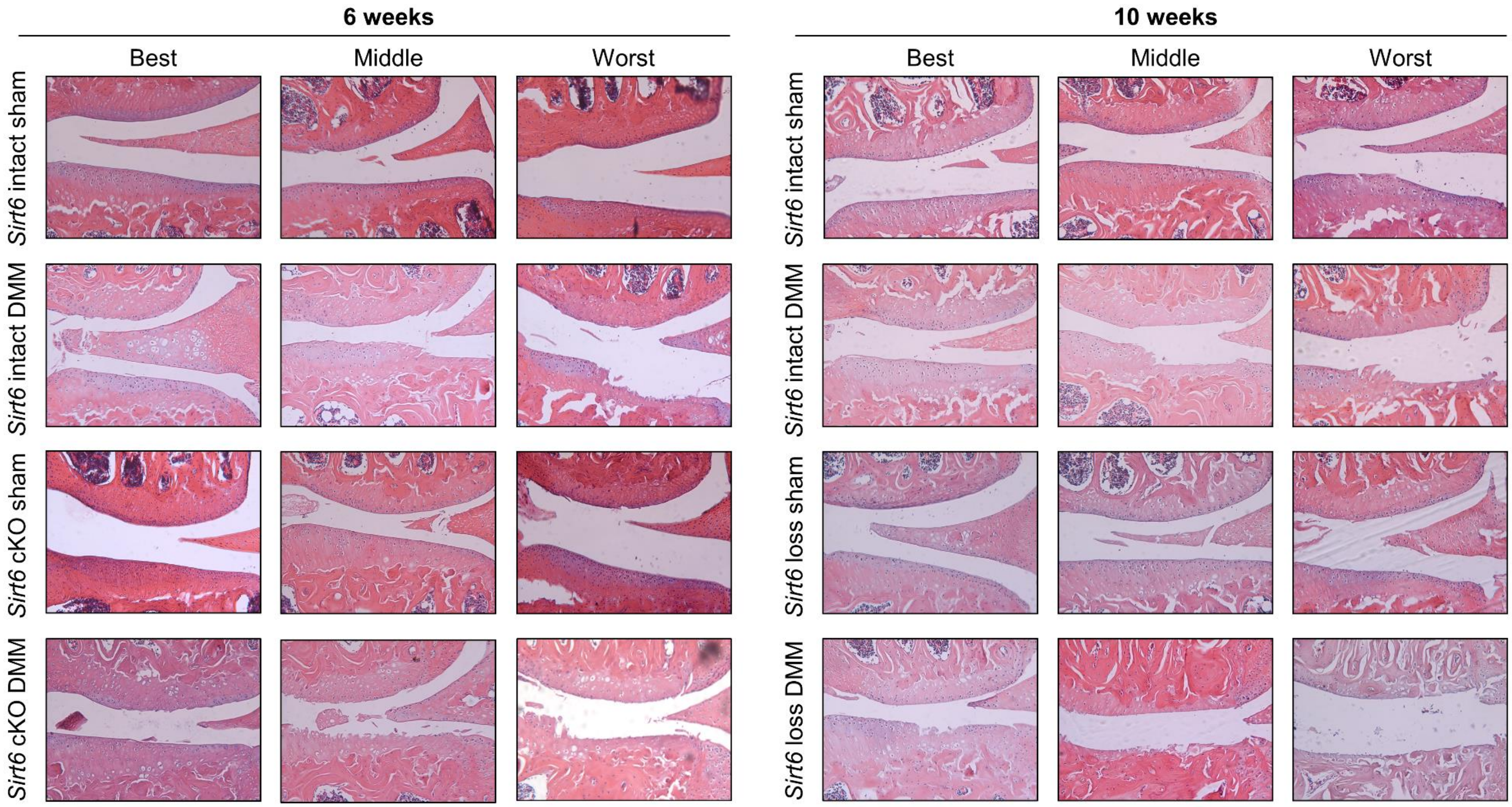

Supplementary Figure 2. Representative images of H&E stained mid coronal sections showing the MTP and MFC from all sham and DMM groups at 6- and 10-weeks post-surgery. Representative examples of the best (minimal damage), middle (average damage) and worst (maximal damage) joints for each genotype are presented.

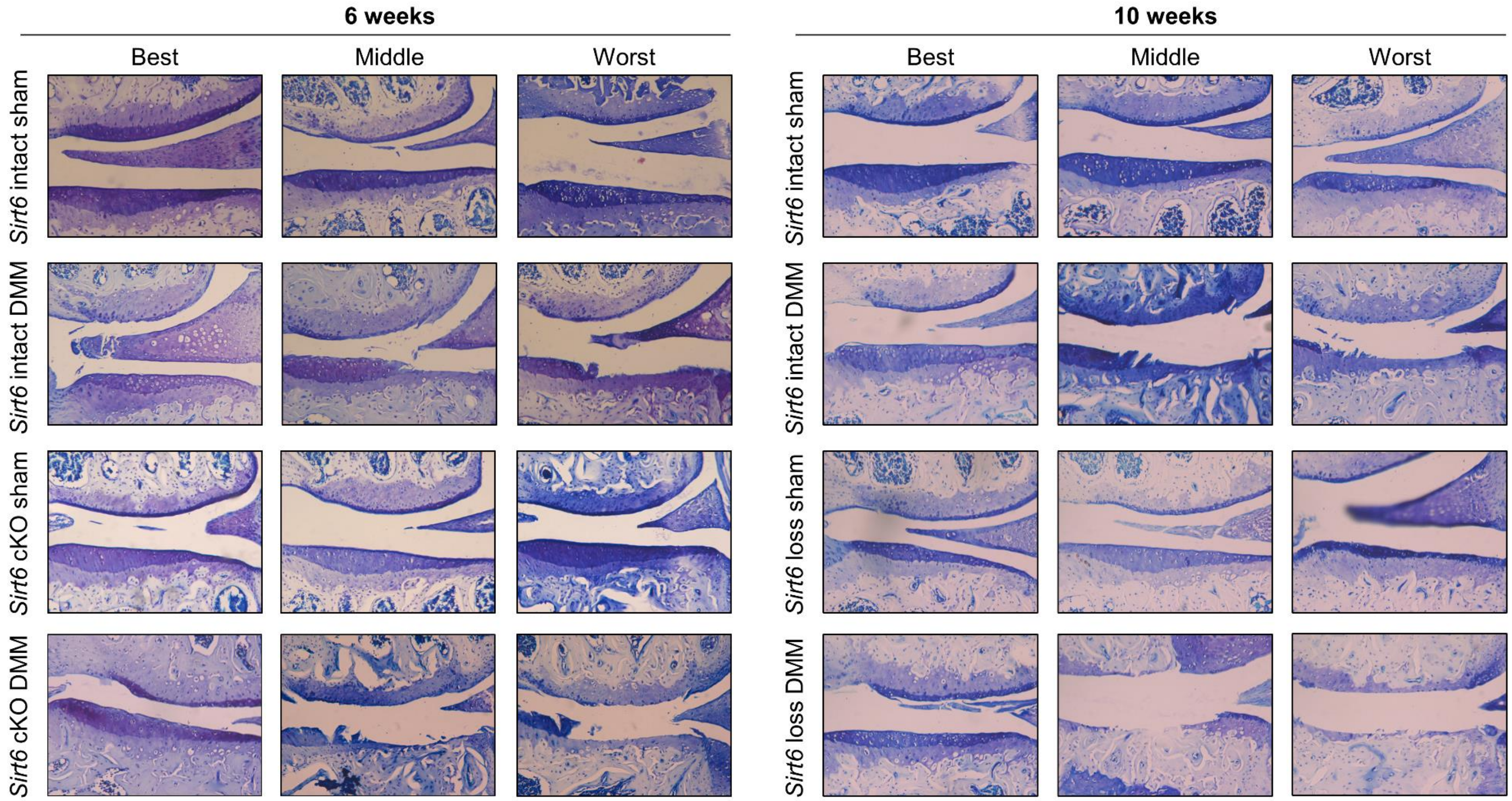

Supplementary Figure 3. Representative images of toluidine blue stained mid coronal sections showing the MTP and MFC from all sham and DMM groups at 6- and 10-weeks post-surgery. Representative examples of the best (minimal damage), middle (average damage) and worst (maximal damage) joints for each genotype are presented.

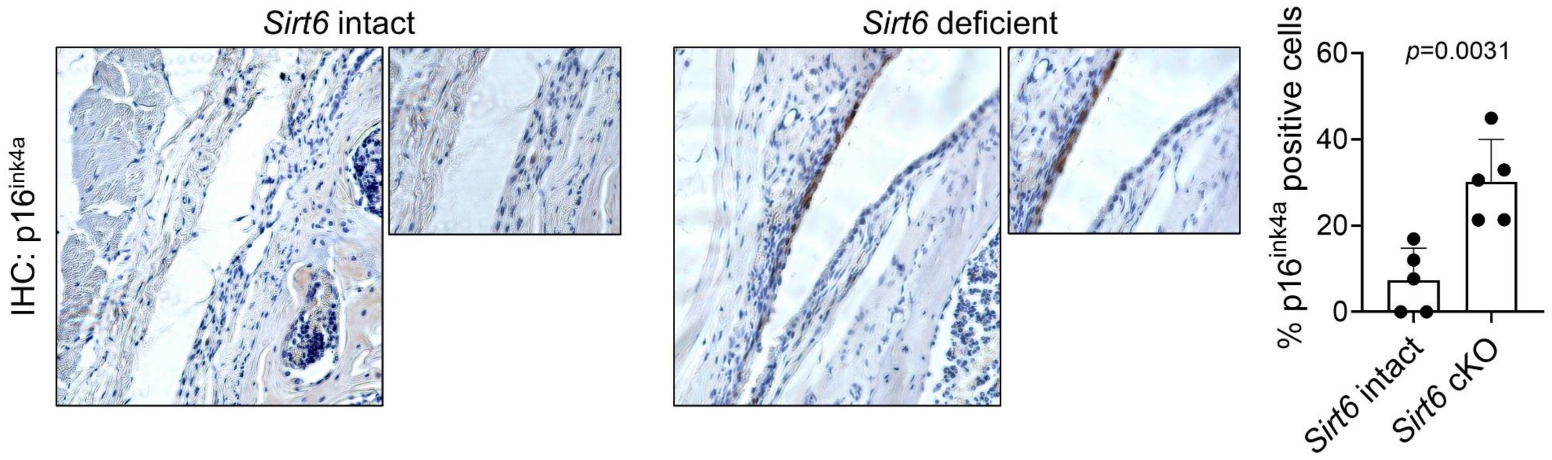

Supplementary Figure 4. Immunohistochemistry to detect p16^{ink4a} levels was performed on midcoronal sections from 18-month-old *Sirt6* intact controls and *Sirt6* deficient mice (n=5) and percentage p16^{ink4a} positively stained cells in the synovium were quantified. Individual data points are presented with mean ± standard deviation (SD). Significant differences between groups were detected by unpaired *t*-test. Exact *p*-values are presented.

BMJ Publishing Group Limited (BMJ) disclaims all liability and responsibility arising from any reliance placed on this supplemental material which has been supplied by the author(s)

**Parameter**	***Sirt6* intact sham** Mean (SD)	***Sirt6* intact DMM** Mean (SD)	***Sirt6* cKO sham** Mean (SD)	***Sirt6* cKO DMM** Mean (SD)	***Sirt6* intact sham vs *Sirt6* intact DMM** *p*-value	***Sirt6* cKO sham vs *Sirt6* cKO DMM** *p*-value	***Sirt6* intact DMM vs *Sirt6* cKO DMM** *p*-value
Art. Cart. Area (μm^2)	52,497.6 (15,741)	57,148 (15,437)	55,403 (18,733)	35,400 (16,368)	0.5074	**0.0151**	**0.0015**
Art. Cart. Thickness (µm)	44.21 (11.45)	44.15 (12.71)	42.61 (14.40)	26.68 (11.18)	0.9933	**0.0093**	**0.0009**
Calc. Cart. Area (μm^2)	45,907 (5,868)	50,124 (11,798)	48,187 (10.231)	39,322 (7,599)	0.3586	**0.0299**	**0.0096**
Calc. Cart. Thickness (µm)	37.99 (4.65)	34.74 (6.86)	38.75 (4.12)	33.118 (8.28)	0.4201	0.0754	0.5820
SCBP Area (μm^2)	82,431 (18,450)	83,778 (27,021)	75,252 (17,406)	79,721 (27,065)	0.9734	0.6681	0.8674
SCBP Thickness (µm)	55.52 (5.08)	54.00 (13.46)	61.12 (19.04)	60.87 (20.36)	0.2973	0.9774	0.4020

Supplementary Table 2. Histomorphometric analysis of *Sirt6* intact and *Sirt6* deficiency mice receiving DMM or sham surgery 6 weeks post-DMM surgery (lateral side). Histomorphometry measurements of articular cartilage (Art. cart) thickness and area, calcified cartilage (Calc. cart) thickness and area, and subchondral bone plate (SCBP) thickness and area were analyzed from mid coronal sections of mouse limbs (lateral tibial plateau) from *Sirt6* intact and *Sirt6* cKO mice receiving DMM or sham surgery 6 weeks post-DMM surgery. *Sirt6* intact sham: n=8; *Sirt6* intact DMM: n=14; *Sirt6* cKO sham: n=9; *Sirt6* cKO DMM: n=13. Results are presented as mean ± standard deviation (SD). Significant differences between groups were detected by Mann-Whitney test comparing sham and DMM groups for each genotype. Exact *p*-values are presented.

Parameter	***Sirt6*** **intact sham** Mean (SD)	***Sirt6*** **intact DMM** Mean (SD)	***Sirt6*** **cKO sham** Mean (SD)	***Sirt6*** **cKO DMM** Mean (SD)	***Sirt6*** **intact sham vs** ***Sirt6*** **intact DMM** *p*-value	***Sirt6*** **cKO sham vs** ***Sirt6*** **cKO DMM** *p*-value	***Sirt6*** **intact DMM vs** ***Sirt6*** **cKO DMM** *p*-value
Art. Cart. Area (µm²)	52,893 (17,890)	55,705 (20,124)	48,844 (17,849)	30,828 (18,901)	0.7439	0.0628	**0.0020**
Art. Cart. Thickness (µm)	43.78 (13.78)	43.67 (12.40)	36.56 (11.1)	22.61 (14.77)	0.9845	0.0541	**0.0003**
Calc. Cart. Area (µm²)	39,270 (7,969)	44,754 (8,081)	59,713 (5,589)	36,117 (10,547)	0.1344	**<0.0001**	**0.0194**
Calc. Cart. Thickness (µm)	33.80 (6.54)	36.78 (6.70)	38.48 (3.86)	25.97 (10.54)	0.3182	**0.0023**	**0.0032**
SCBP Area (µm²)	69,716 (14,786)	77,555 (20,283)	91,305 (26,452)	79,080 (27,260)	0.3475	0.3664	0.8650
SCBP Thickness (µm)	55.16 (8.88)	54.27 (13.01)	56.04 (12.39)	47.61 (14.78)	0.8651	0.2382	0.2076

Supplementary Table 3. Histomorphometric analysis of *Sirt6* intact and *Sirt6* deficiency mice receiving DMM or sham surgery 10 weeks post-surgery (lateral side). Histomorphometry measurements of articular cartilage (Art. cart) thickness and area, calcified cartilage (Calc. cart) thickness and area, and subchondral bone plate (SCBP) thickness and area were analyzed from mid coronal sections of mouse limbs (lateral tibial plateau) from *Sirt6* intact and *Sirt6* cKO mice receiving DMM or sham surgery 10 weeks post-DMM surgery. *Sirt6* intact sham: n=8; *Sirt6* intact DMM: n=15; *Sirt6* cKO sham: n=6; *Sirt6* cKO DMM: n=14. Results are presented as mean ± standard deviation (SD). Significant differences between groups were detected by Mann-Whitney test comparing sham and DMM groups for each genotype. Exact *p*-values are presented.

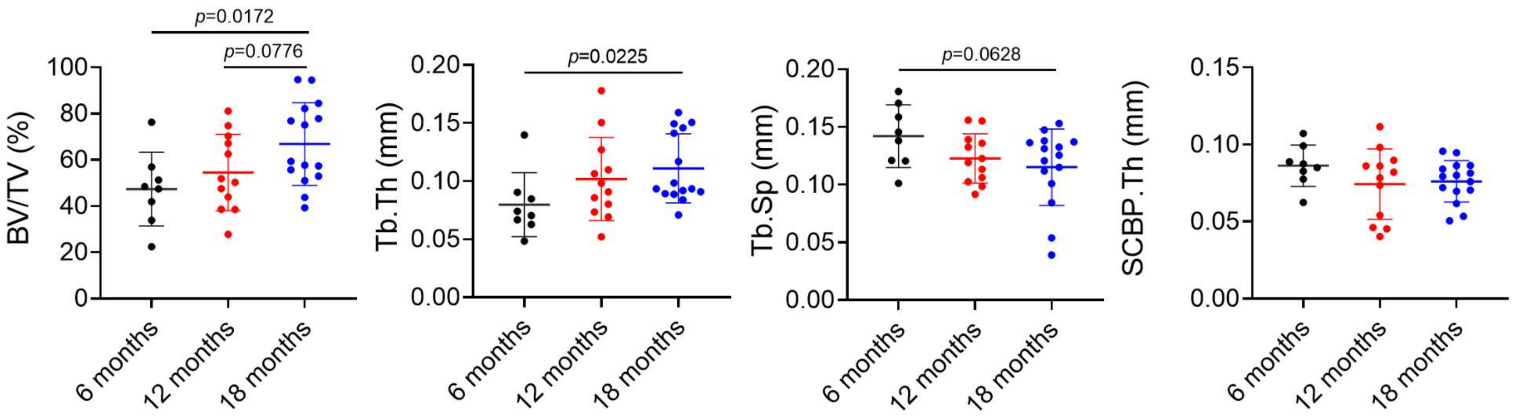

Supplementary Figure 5. Subchondral bone changes during aging. MicroCT analysis of bone changes (BV/TV, Tb.Th, Tb.Sp, SCBP.Th) in *Sirt6* intact sham control mouse limbs aged 6 months of age, and Sirt6 intact mouse limbs at 12 months, and 18 months of age. Individual data points are presented with mean ± standard deviation (SD). Exact *p*-values are presented.

Collins JA, *et al. Ann Rheum Dis* 2023; 82:1464–1473. doi: 10.1136/ard-2023-224385

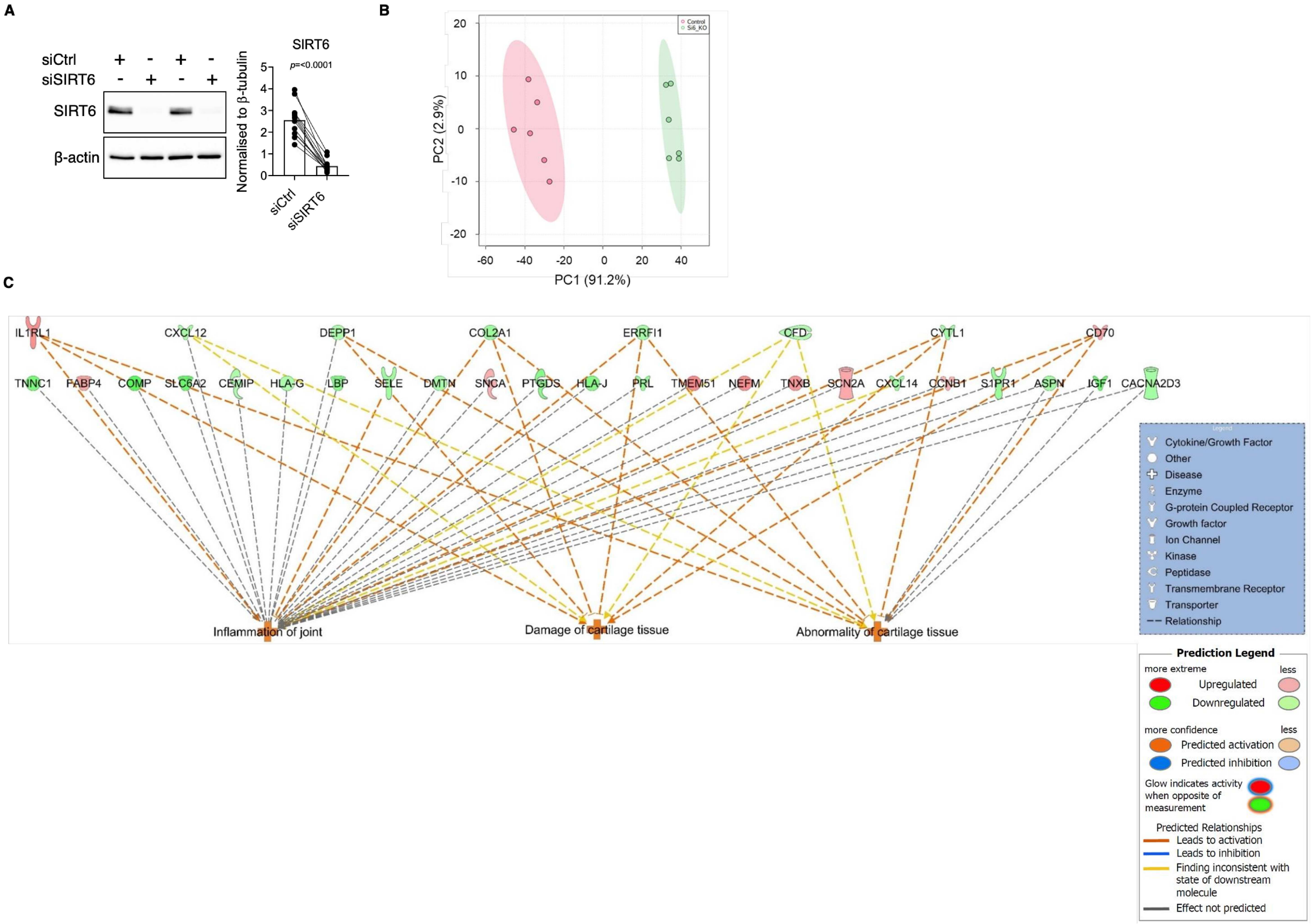
A
siCtrl + - + -
siSIRT6 - + - +
SIRT6
β-actin
SIRT6
p=<0.0001
Normalised to β-tubulin
siCtrl
siSIRT6
B
Control
Si6_KO
PC2 (2.9%)
PC1 (91.2%)
C
IL1RL1
CXCL12
DEPP1
COL2A1
ERRFI1
CFD
CYTL1
CD70
TNNC1
FABP4
COMP
SLC6A2
CEMIP
HLA-G
LBP
SELE
DMTN
SNCA
PTGDS
HLA-J
PRL
TMEM51
NEFM
TNXB
SCN2A
CXCL14
CCNB1
S1PR1
ASPN
IGF1
CACNA2D3
Inflammation of joint
Damage of cartilage tissue
Abnormality of cartilage tissue
Cytokine/Growth Factor
Other
Disease
Enzyme
G-protein Coupled Receptor
Growth factor
Ion Channel
Kinase
Peptidase
Transmembrane Receptor
Transporter
Relationship
Prediction Legend
more extreme
less
Upregulated
Downregulated
more confidence
less
Predicted activation
Predicted inhibition
Glow indicates activity when opposite of measurement
Predicted Relationships
Leads to activation
Leads to inhibition
Finding inconsistent with state of downstream molecule
Effect not predicted

314

Supplementary Figure 6. (**A**) Primary human chondrocytes were nucleofected with siRNA to *Sirt6* or a scrambled siRNA control (72 hrs.) to validate *Sirt6* knockdown. Cells were harvested and immunoblotting for total SIRT6 was performed. Presented immunoblots are representative and protein bands were normalized to β actin. Individual data points are presented with mean ± standard deviation (SD). Significant differences were detected by paired *t*-test and exact *p*-values are presented. (**B**) principal component analysis (PCA) was performed on our RNA-sequencing dataset and showed that groups were clustered by treatment. (**C**) Regulatory effects analysis was conducted using the Ingenuity Pathway Analysis (IPA) tool to show the effect of *Sirt6* deficiency on downstream functions that involve the differentially expressed genes in our RNA sequencing dataset.

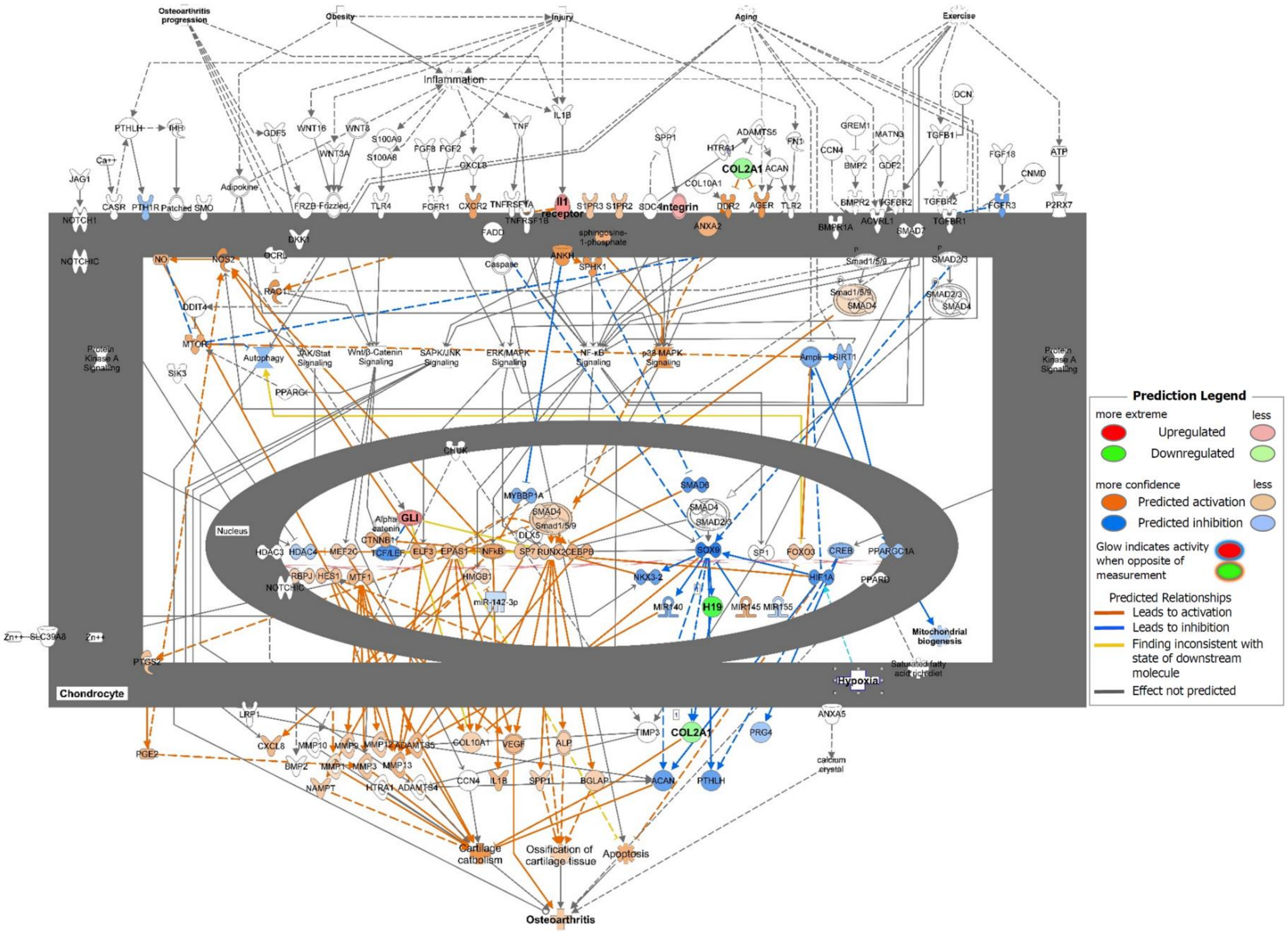

Osteoarthritis progression
Obesity
Injury
Aging
Exercise
Inflammation
DCN
PTHLH
IHH
GDF5
WNT16
WNT8
S100A9
S100A8
WNT3A
FGF8
FGF2
CXCL8
TNF
IL1B
SPP1
HTRA1
ADAMTS5
FN1
COL2A1
ACAN
COL10A1
GREM1
MATN3
TGFB1
CCN4
BMP2
GDF2
FGF18
ATP
CNMD
JAG1
Ca++
Adipokine
NOTCH1
CASR
PTH1R
Patched
SMO
FRZB
Frizzled
TLR4
FGFR1
CXCR2
TNFRSF1A
TNFRSF1B
Il1 receptor
S1PR3
S1PR2
SDC4
Integrin
DDR2
AGER
TLR2
BMPR2
TGFBR2
FGFR3
P2RX7
BMPR1A
ACVRL1
SMAD7
TGFBR1
ANXA2
FADD
DKK1
sphingosine-1-phosphate
NOTCHIC
NO
NOS2
OCRL
Caspase
ANKH
SPHK1
Smad1/5/9
SMAD2/3
SMAD4
RAC1
DDIT4
MTOR
Protein Kinase A Signaling
Autophagy
JAK/Stat Signaling
Wnt/β-Catenin Signaling
SAPK/JNK Signaling
ERK/MAPK Signaling
NF-κB Signaling
p38 MAPK Signaling
Ampk
SIRT1
SIK3
PPARG
CHUK
MYBBP1A
SMAD6
Alpha catenin
GLI
CTNNB1
DLX5
Nucleus
HDAC3
HDAC4
MEF2C
TCF/LEF
ELF3
EPAS1
NFKB
SP7
RUNX2
CEBPB
SOX9
SP1
FOXO3
CREB
PPARGC1A
RBPJ
HES1
MTF1
HMGB1
NKX3-2
HIF1A
PPARD
miR-142-3p
MIR140
H19
MIR145
MIR155
Mitochondrial biogenesis
Zn++
SLC39A8
PTGS2
Saturated fatty acid rich diet
Hypoxia
Chondrocyte
LRP1
ANXA5
PGE2
CXCL8
MMP10
MMP9
MMP12
ADAMTS5
COL10A1
VEGF
ALP
TIMP3
COL2A1
PRG4
BMP2
MMP1
MMP3
MMP13
NAMPT
HTRA1
ADAMTS4
CCN4
IL1B
SPP1
BGLAP
ACAN
PTHLH
calcium crystal
Cartilage catabolism
Ossification of cartilage tissue
Apoptosis
Osteoarthritis
Prediction Legend
more extreme
less
Upregulated
Downregulated
more confidence
less
Predicted activation
Predicted inhibition
Glow indicates activity when opposite of measurement
Predicted Relationships
Leads to activation
Leads to inhibition
Finding inconsistent with state of downstream molecule
Effect not predicted

Supplementary Figure 7. Ingenuity Pathway Analysis (IPA) predicting downstream effects implicated in the OA disease process in chondrocytes depleted of *SIRT6* in our RNA-sequencing dataset. Predicted analysis demonstrates that deficiency of *SIRT6* would lead to activation (upregulated) of cartilage catabolism, ossification of cartilage tissue, apoptosis, and ultimately osteoarthritis.

Collins JA, *et al. Ann Rheum Dis* 2023; 82:1464–1473. doi: 10.1136/ard-2023-224385

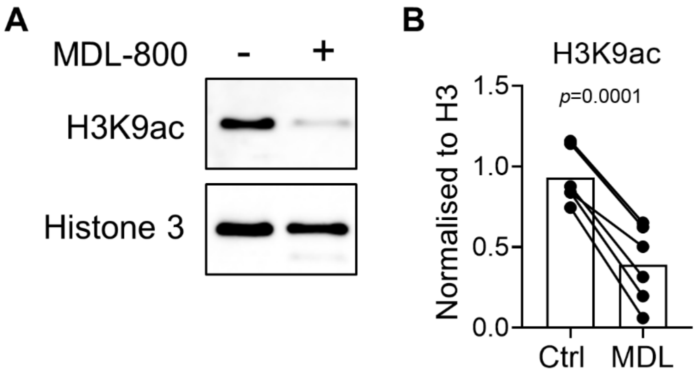

Supplementary Figure 8. (**A**) Primary human chondrocytes were treated with the small molecule SIRT6 activator, MDL-800, for 24 hrs. (12.5 µM) and histones were isolated prior to immunoblotting with antibodies to acetylated H3K9 (H3K9ac) as an inverse marker of SIRT6 activity. Immunoblots are representative of n=6 independent experiments. (**B**) Densitometric analysis from H3K9ac immunoblots. Data was normalized to Histone 3 which was used as a loading control. Data is presented as mean ± standard deviation (SD) compared to untreated control values. Paired *t*-test was used to test for significant differences between groups. Exact *p* values are presented.

References

1. Ma HL, Blanchet TJ, Peluso D, Hopkins B, Morris EA, Glasson SS. Osteoarthritis severity is sex dependent in a surgical mouse model. Osteoarthritis Cartilage. 2007;15(6):695-700.
2. Loeser RF, Kelley KL, Armstrong A, Collins JA, Diekman BO, Carlson CS. Deletion of c-Jun N-terminal Kinase Enhances Senescence in Joint Tissues and Increases the Severity of Age-Related Osteoarthritis in Mice. Arthritis Rheumatol. 2020.
3. Collins JA, Kapustina M, Bolduc JA, Pike JFW, Diekman BO, Mix K, et al. Sirtuin 6 (SIRT6) regulates redox homeostasis and signaling events in human articular chondrocytes. Free Radic Biol Med. 2021;166:90-103.
4. Loeser RF, Coryell PR, Armstrong AR, Collins JA, Gopalakrishnan P, McDermott KA, et al. Overexpression of Peroxiredoxin 3 in Cartilage Reduces the Severity of Age-Related Osteoarthritis But Not Surgically Induced Osteoarthritis in Mice. ACR Open Rheumatol. 2022;4(5):441-6.
5. Glasson SS, Blanchet TJ, Morris EA. The surgical destabilization of the medial meniscus (DMM) model of osteoarthritis in the 129/SvEv mouse. Osteoarthritis Cartilage. 2007;15(9):1061-9.
6. Fang H, Huang L, Welch I, Norley C, Holdsworth DW, Beier F, et al. Early Changes of Articular Cartilage and Subchondral Bone in The DMM Mouse Model of Osteoarthritis. Sci Rep. 2018;8(1):2855.
7. Loeser RF, Olex AL, McNulty MA, Carlson CS, Callahan M, Ferguson C, et al. Disease progression and phasic changes in gene expression in a mouse model of osteoarthritis. PLoS One. 2013;8(1):e54633.
8. Zhang QS, Eaton GJ, Diallo C, Freeman TA. Stress-Induced Activation of Apoptosis Signal-Regulating Kinase 1 Promotes Osteoarthritis. J Cell Physiol. 2016;231(4):944-53.
9. Kim YH, Kang JS. Micro-computed tomography evaluation and pathological analyses of female rats with collagen-induced arthritis. J Vet Sci. 2015;16(2):165-71.
10. Loeser RF, Olex AL, McNulty MA, Carlson CS, Callahan MF, Ferguson CM, et al. Microarray analysis reveals age-related differences in gene expression during the development of osteoarthritis in mice. Arthritis Rheum. 2012;64(3):705-17.
11. Rowe MA, Harper LR, McNulty MA, Lau AG, Carlson CS, Leng L, et al. Reduced Osteoarthritis Severity in Aged Mice With Deletion of Macrophage Migration Inhibitory Factor. Arthritis Rheumatol. 2017;69(2):352-61.
12. McNulty MA, Loeser RF, Davey C, Callahan MF, Ferguson CM, Carlson CS. A Comprehensive Histological Assessment of Osteoarthritis Lesions in Mice. Cartilage. 2011;2(4):354-63.
13. Armstrong AR, Carlson CS, Rendahl AK, Loeser RF. Optimization of histologic grading schemes in spontaneous and surgically-induced murine models of osteoarthritis. Osteoarthritis Cartilage. 2021;29(4):536-46.
14. Muehleman C, Bareither D, Huch K, Cole AA, Kuettner KE. Prevalence of degenerative morphological changes in the joints of the lower extremity. Osteoarthritis Cartilage. 1997;5(1):23-37.

15. Collins JA, Wood ST, Nelson KJ, Rowe MA, Carlson CS, Chubinskaya S, et al. Oxidative Stress Promotes Peroxiredoxin Hyperoxidation and Attenuates Pro-survival Signaling in Aging Chondrocytes. J Biol Chem. 2016;291(13):6641-54.
16. Collins JA, Wood ST, Bolduc JA, Nurmalasari NPD, Chubinskaya S, Poole LB, et al. Differential peroxiredoxin hyperoxidation regulates MAP kinase signaling in human articular chondrocytes. Free Radic Biol Med. 2019;134:139-52.
17. Michishita E, McCord RA, Berber E, Kioi M, Padilla-Nash H, Damian M, et al. SIRT6 is a histone H3 lysine 9 deacetylase that modulates telomeric chromatin. Nature. 2008;452(7186):492-6.
18. Hu S, Liu H, Ha Y, Luo X, Motamedi M, Gupta MP, et al. Posttranslational modification of Sirt6 activity by peroxynitrite. Free Radic Biol Med. 2015;79:176-85.
19. Mao Z, Hine C, Tian X, Van Meter M, Au M, Vaidya A, et al. SIRT6 promotes DNA repair under stress by activating PARP1. Science. 2011;332(6036):1443-6.
20. Pan H, Guan D, Liu X, Li J, Wang L, Wu J, et al. SIRT6 safeguards human mesenchymal stem cells from oxidative stress by coactivating NRF2. Cell Res. 2016;26(2):190-205.
21. Kim HS, Xiao C, Wang RH, Lahusen T, Xu X, Vassilopoulos A, et al. Hepatic-specific disruption of SIRT6 in mice results in fatty liver formation due to enhanced glycolysis and triglyceride synthesis. Cell Metab. 2010;12(3):224-36.
22. Qin K, Zhang N, Zhang Z, Nipper M, Zhu Z, Leighton J, et al. SIRT6-mediated transcriptional suppression of Txnip is critical for pancreatic beta cell function and survival in mice. Diabetologia. 2018;61(4):906-18.
23. Mortazavi A, Williams BA, McCue K, Schaeffer L, Wold B. Mapping and quantifying mammalian transcriptomes by RNA-Seq. Nat Methods. 2008;5(7):621-8.
24. Liao Y, Smyth GK, Shi W. featureCounts: an efficient general purpose program for assigning sequence reads to genomic features. Bioinformatics. 2014;30(7):923-30.
25. Kim D, Paggi JM, Park C, Bennett C, Salzberg SL. Graph-based genome alignment and genotyping with HISAT2 and HISAT-genotype. Nat Biotechnol. 2019;37(8):907-15.
26. Love MI, Huber W, Anders S. Moderated estimation of fold change and dispersion for RNA-seq data with DESeq2. Genome Biol. 2014;15(12):550.
27. Watanabe H, Ishii H, Takahashi K, Takai S, Ozawa H. Suitable reference gene selection for gene expression studies in knee osteoarthritis synovium using quantitative PCR analysis. Connect Tissue Res. 2018;59(4):356-68.
28. Livak KJ, Schmittgen TD. Analysis of relative gene expression data using real-time quantitative PCR and the 2(-Delta Delta C(T)) Method. Methods. 2001;25(4):402-8.
